# Gut microbiota markers associated with obesity and overweight in Italian adults

**DOI:** 10.1038/s41598-021-84928-w

**Published:** 2021-03-09

**Authors:** Vanessa Palmas, Silvia Pisanu, Veronica Madau, Emanuela Casula, Andrea Deledda, Roberto Cusano, Paolo Uva, Sarah Vascellari, Andrea Loviselli, Aldo Manzin, Fernanda Velluzzi

**Affiliations:** 1 Microbiology and Virology Unit, Department of Biomedical Sciences, Azienda Ospedaliero-Universitaria Cagliari, University of Cagliari, Strada Statale 554- bivio Sestu, 09042 Monserrato, Cagliari Italy; 2 Department of Medical Sciences and Public Health, University of Cagliari, Cagliari, Italy; 3 CRS4, Science and Technology Park Polaris, Piscina Manna, Pula, Cagliari Italy

**Keywords:** Microbiology, Microbial communities, Microbiome

## Abstract

In the present study, we characterized the distinctive signatures of the gut microbiota (GM) from overweight/obese patients (OB), and normal-weight controls (NW), both of Sardinian origin. Fecal bacterial composition of 46 OB patients (BMI = 36.6 ± 6.0; F/M = 40/6) was analyzed and compared to that of 46 NW subjects (BMI = 21.6 ± 2.1; F/M = 41/5), matched for sex, age and smoking status, by using 16S rRNA gene sequencing on MiSeq Illumina platform. The gut microbial community of OB patients exhibited a significant decrease in the relative abundance of several Bacteroidetes taxa (i.e. Flavobacteriaceae, Porphyromonadaceae, Sphingobacteriaceae, *Flavobacterium, Rikenella* spp*., Pedobacter* spp*., Parabacteroides* spp., *Bacteroides* spp.) when compared to NW; instead, several Firmicutes taxa were significantly increased in the same subjects (Lachnospiraceae, Gemellaceae, Paenibacillaceae, Streptococcaceae, Thermicanaceae, *Gemella*, *Mitsuokella*, *Streptococcus*, *Acidaminococcus* spp., *Eubacterium* spp., *Ruminococcus* spp., *Megamonas* spp., *Streptococcus*, *Thermicanus*, *Megasphaera* spp. and *Veillonella* spp*.*). Correlation analysis indicated that body fatness and waist circumference negatively correlated with Bacteroidetes taxa, while Firmicutes taxa positively correlated with body fat and negatively with muscle mass and/or physical activity level. Furthermore, the relative abundance of several bacterial taxa belonging to Enterobacteriaceae family, known to exhibit endotoxic activity, was increased in the OB group compared to NW. The results extend our knowledge on the GM profiles in Italian OB, identifying novel taxa linking obesity and intestine.

## Introduction

Obesity is one of the major health problems in the world due to the rapid increase in its prevalence and the emergence of metabolic co-morbidities observed since the mid-twentieth century. In Italy, in 2017 more than a third of the population (35.4%) aged 18 and over was overweight, while a little more than one in ten (10.5%) was obese. In line with these findings, the prevalence of obesity in Sardinia has more than tripled in the last 30 years^1,2^. Defined as a chronic disease, obesity is associated with increased mortality and represents a risk factor for the development of various diseases, including cardiovascular diseases, type 2 diabetes mellitus (T2DM), musculoskeletal disorders, various form of cancer, asthma, pulmonary embolism, gallbladder disease and metabolic syndrome^3–5^. The multifactorial etiology of obesity includes genetic, behavioral, socioeconomic, and environmental components^6^; however, the putative role of the gut microbiota (GM) disfunction (dysbiosis) in the pathogenesis of the disease has recently emerged. The GM is the community of microorganisms (bacteria, archaea, viruses, eukaryotes) colonizing the digestive tract^7^, and its alterations has been linked aside from functional gastrointestinal disorders, to obesity and other metabolic disorders, including T2DM and non-alcoholic fatty liver disease (NAFLD); furthermore, it could contribute to the development of metabolic syndrome (MS)^5,8^. Several mechanisms have been proposed to explain the potential role of GM in obesity development. Among them, the fermentation of non-digestible dietary polysaccharides by intestinal bacteria, resulting in the production of short-chain fatty acids (SCFAs), which can induce lipogenesis in the liver and triglycerides accumulation in host adipocytes. In addition, the GM can trigger a chronic low-grade inflammation (metabolic endotoxemia), due to the systemic translocation of the Gram-negative bacteria lipopolysaccharides (LPS)^9,10^. Metabolic endotoxemia, as well as lower microbial diversity, are presumed to be linked to the etiology of MS and its consequences^5^. The latter is a cluster of conditions including abdominal obesity, hyperglycemia, hypertension, insulin resistance, and dyslipidemia, whose link with GM composition has been confirmed by animal and human studies^11^. However, regarding the association between specific bacterial taxa and these pathological processes, only a few studies have examined the GM composition in the obese Italian adult population^12–14^, and few data are available regarding patients with MS^15^. This makes further scientific investigations necessary in order to identify a microbial pattern associated with metabolic diseases. The identification of microbial composition is, indeed, a first and relevant step towards the development of strategies for early diagnosis, monitoring of disease progression and its treatment. In the present study, our *focus* was to characterize the distinctive signatures of the GM from overweight/obese patients of Sardinian origin, compared to healthy controls; in addition, to verify, within the obese cohort, the extent of gut dysbiosis in patients with MS.

## Results

### Subjects

Clinical characteristics of the study groups are shown in Table 1. Remarkably, the fraction of women was prevalent in each study cohort, but no statistically significant differences were observed in terms of age and gender. Overall, the analysis of clinical data showed that the OB group diverged significantly from healthy controls in terms of BMI, waist circumference and various lifestyle factors. As regard to lifestyle data, the nutritional anamnesis highlighted in patients an excessive consumption of sugary drinks, sweets, industrial food, and a low consumption of fruit, vegetables, and pulses, as well as the absence of whole grains. In line with these findings, the analysis of the food diaries pointed out an excess of simple carbohydrates, of total lipids and saturated fatty acids and a lack of fibers intake; on the other hand, control subjects followed, as expected, a healthier diet, in terms of macronutrients quality, and caloric and fiber intake, which were more in line with National Recommended Energy and Nutrient Intake Levels (LARN) guidelines^16^. Noteworthy, patients had an excessive intake of saturated lipids (38% of the total lipid intake for OB), significantly greater than that of NW (27.8% of the total lipid intake) and, contrariwise, a low daily intake of fiber (15 g/day), significantly lower than that of NW (20.0 g/day). The Mediterranean Diet Score (MedDietScore) highlighted good compliance to Mediterranean Diet (MD) in NW (33.4 ± 4.1), while it was lower in OB (28 ± 5), although this difference was not statistically significant (*p* = 0.106). The level of physical activity was low in the group of patients and significantly reduced compared to NW, who, in contrast, engaged in a medium–high level of physical activity. By stratifying the OB group for the presence of metabolic syndrome, the anthropometric and lifestyle data of overweight and obese patients with MS (OMS) and without MS (OWMS) almost reflected those of non-stratified OB; on the other hand, no statistically difference was observed between OMS and OWMS for each clinical and metabolic alterations, except, as expected, for the prevalence of cardiometabolic risk factors (dyslipidemia, alteration in glucose metabolism and hypertension) (see Supplementary Table S1 online).

**Table 1:** Clinical characteristics of study participants.

	NW	OB	*p*
N	46	46	
Age (M ± SD)	49 ± 11	50 ± 12	0.345
Female (n, %)	40, 87.0	40, 87.0	1.000
**Anthropometric data**			
BMI (kg/m^2^) (M ± SD)	21.6 ± 2.1	36.0 ± 6.0	**9.9 × 10**^**−5**^
Fat mass (kg) (M ± SD)		39.1 ± 11.9	
Fat mass (%) (M ± SD)		42.3 ± 5.7	
Muscle mass (Kg) (M ± SD)		48.5 ± 11.3	
Waist circumference (cm) (M ± SD)	73.7 ± 5.7	111 ± 15	**7 × 10**^**−6**^
Overweight (N, %)		5, 11	
Obesity Class I (N; %)		17, 37	
Obesity Class II (N; %)		17, 37	
Obesity Class III (N, %)		7, 15	
**Lifestyle factors**			
Smoking status Yes (n, %)	8, 17.8	12, 26	0.339
Alcohol consumption None (n, %)	10, 21.7	21, 46	**0.015**
Alcohol consumption Rare (n, %)	24, 52.2	17, 37	0.142
Alcohol consumption Moderate (n, %)	12, 26.1	8, 17	0.312
Daily caloric intake, kcal (M ± SD)	1467.8 ± 162.3	1810 ± 627	**1.563 × 10**^**−8**^
Daily carbohydrates intake, % (M ± SD)	50.9 ± 3	50 ± 7	**8 × 10**^**−6**^
Daily lipids intake, % (M ± SD)	27.2 ± 4.3	33 ± 6	**0.006**
Daily saturated lipids intake/total lipids intake, % (M ± SD)	27.8 ± 4.3	38 ± 7	**0.022**
Daily proteins intake, grams (M ± SD)	62.3 ± 8.8	78 ± 43	**0.001**
Daily fiber intake, grams (M ± SD)	20.0 ± 3.2	15 ± 8	**0.018**
MedDietScore (M ± SD)	33.4 ± 4.1	28 ± 5	0.106
IPAQ METs/week (M ± SD)	2604.8 ± 135.4	662 ± 28	**1 × 10**^**−6**^
**Dyslipidemia**			
Yes (n, %)	0	12, 26	
Alteration in glucose metabolism*			
Yes (n, %)	0	32, 70	
**Hypertension**			
Yes, %	0	11, 24	

*impaired Fasting Plasma Glucose (FPG) between 100 and 125 mg/dl (6.9 mmol/l), impaired glucose tolerance (IGT) if 2 h post-OGTT plasma glucose was 140–199 mg/dl (7.8–11.0 mmol/l), T2DM if FPG was ≥ 126 mg/dl (≥ 7 mmol/l) on two days apart, or if 2 h post-OGTT plasma glucose was ≥ 200 mg/dl (≥ 11.1 mmol/l). The statistical significance was evaluated by *t* test for independent samples for continuous variables and by Pearson’s chi-squared test for categorical variables. Bold values denote statistical significance (*p* < 0.05). NW = healthy normal-weight controls, OB = obese and overweight patients.

## Gut microbiota analysis

### Alpha and beta diversity analysis

**Figure 1:**
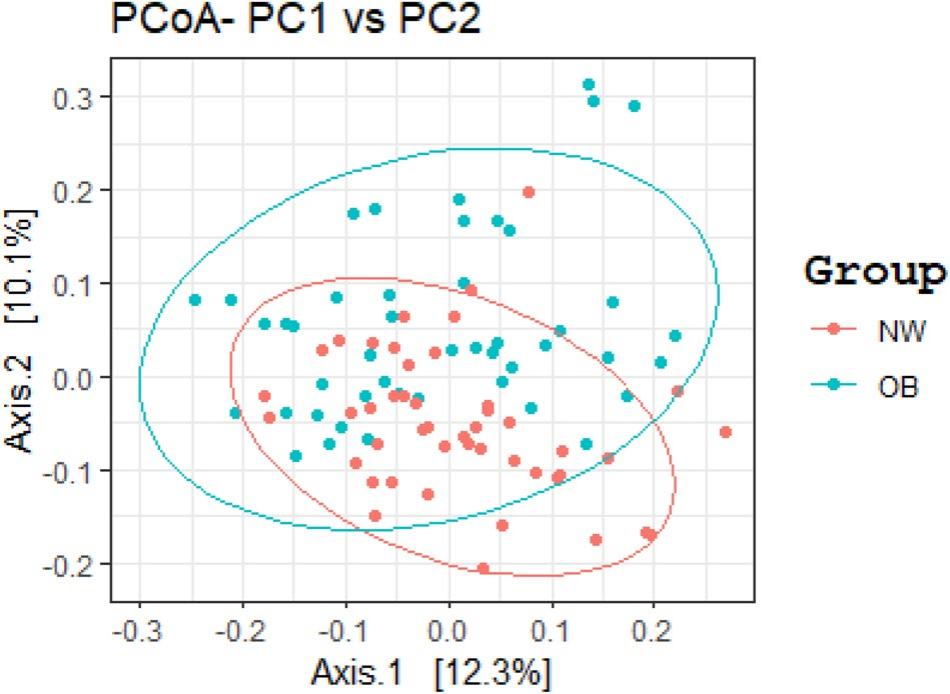
GM beta diversity analysis of OB patients compared to NW. Principal Coordinates Analysis (PCoA) based on Bray–Curtis distance matrix, performed in R software v.3.5.2 (ggplot2 package), showed a marked separation between the GM communities of OB and NW. The statistical significance among the two groups was determined with Permutational Multivariate Analysis of Variance (PERMANOVA) (R-vegan, function adonis), adjusted for sex, age and smoking status (sum of squares = 0.5492, mean of squares = 5.0297, F = 0.0533, R = 0.047, *p* = 0.002). *p* equal to or less than 0.05 was considered statistically significant.

The richness and diversity of the microbial community (alpha diversity) in OB was lower than that in NW, albeit no significant difference in the Shannon index was observed (OB = 2.299 ± 0.338, NW = 2.334 ± 0.175, *p* = 0.833). Similarly, no statistically significant differences in the Shannon index across different BMI categories and between metabolic syndrome discordant obese patient subgroups were observed (*p* = 0.780 and 0.873, respectively) (see Supplementary Tables S2, S3, S4 online). The Principal Coordinates Analysis (PCoA) based on Bray–Curtis distance matrix showed a marked separation between the GM communities of OB and NW (see Fig. 1), confirmed by PERMANOVA analysis, adjusted for sex, age and smoking status, that indicated a significant difference in beta diversity between the two cohorts (sum of squares = 0.5492, mean of squares = 5.0297, F = 0.0533, R = 0.047, *p* = 0.002).

Similarly, PERMANOVA analysis showed a significant segregation between NW, OMS and OWMS cohorts (sum of squares = 0.642, mean of squares = 0.321, F = 2.955, R = 0.007, *p* = 0.007), which persisted only in the comparison between OMS and NW, following the pairwise PERMANOVA test (*p* = 0.030) (see Supplementary Fig. S1 online). Furthermore, although the PERMANOVA test showed significant segregation of the GM across different BMI categories (sum of squares = 0.790; mean of squares = 0.198; F = 1.807; R = 0.077, *p* = 0.025), the same was not confirmed by the pairwise PERMANOVA.

### Gut microbiota composition

A total of 10,356,014 sequencing reads were obtained from the 92 fecal samples, with a mean value of 112,565 (+ /- 20,280 SD) reads generated per patient. The median and range values of each OTU included in the statistical analysis can be found as Supplementary Table S5 online, for OB and NW, respectively.

**Figure 2:**
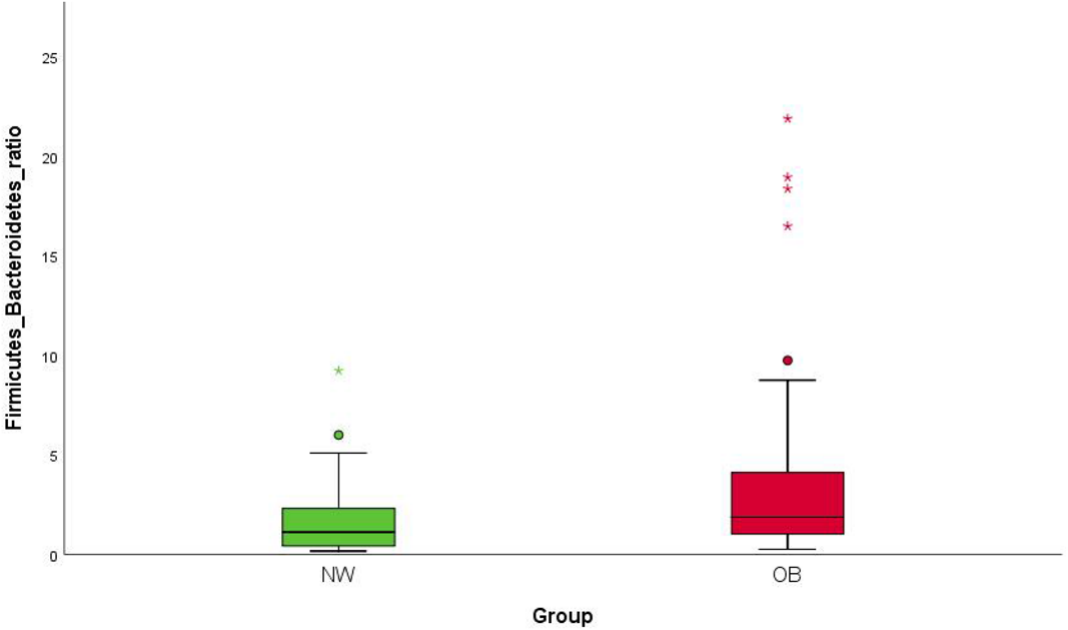
Statistically significant differences in Firmicutes/Bacteroidetes ratio between NW and OB. The statistical significance was calculated by the non- parametric Mann–Whitney test in R software v.3.5.2. Plots were generated in R software v.3.5.2 (ggplot2 package). The Firmicutes/Bacteroidetes ratio was significantly higher in OB, with a value more than twice that of NW (*p* = 0.007; M ± SD: 4.05 ± 5.26 in OB, M ± SD: 1.75 ± 1.82 in NW). *p* value equal to or less than 0.05 was considered statistically significant. NW = normal-weight healthy controls, OB = overweight and obese patients.

The Firmicutes/Bacteroidetes ratio (see Fig. 2) was significantly higher in OB, with a value more than twice that of NW (*p* = 0.007; M ± SD: 4.05 ± 5.26 in OB, M ± SD: 1.75 ± 1.82 in NW). Statistical significance was also maintained when comparison between patients with MS and NW was performed, with an increase in ratio in patients (*p* = 0.025); no significant differences were observed when Firmicutes/Bacteroidetes ratio among different BMI categories was compared (*p* = 0.11) (see Supplementary Tables S6, S7, S8 and Fig. S2 online).

Multivariate analysis on GM composition between OB and NW, adjusted for sex, age and smoking status, showed 44 statistically significant results, which are shown in Table 2. Median and range for each significant result can be found as Supplementary Table S9 online.

**Table 2:** Statistically significant differences in the relative abundance of bacterial taxa between OB and NW.

Phylum	Family	Genus	Species	*q*	Coefficient	↓/↑
Bacteroidetes				0.0249	− 0.1425	↓
Bacteroidetes	Bacteroidaceae	*Bacteroides*	*B. rodentium*	0.0197	− 0.0390	↓
Bacteroidetes	Bacteroidaceae	*Bacteroides*	*B. uniformis*	0.0004	− 0.0731	↓
Bacteroidetes	Flavobacteriaceae			0.0042	− 0.0617	↓
Bacteroidetes	Flavobacteriaceae	*Flavobacterium*		0.0183	− 0.0537	↓
Bacteroidetes	Porphyromonadaceae			0.0107	− 0.0442	↓
Bacteroidetes	Rikenellaceae	*Rikenella*		0.0037	− 0.0128	↓
Bacteroidetes	Rikenellaceae	*Rikenella*	*R. microfusus*	0.0037	− 0.0128	↓
Bacteroidetes	Sphingobacteriaceae			0.0001	− 0.0368	↓
Bacteroidetes	Sphingobacteriaceae	*Pedobacter*		2.92 × 10^**−**9^	− 0.0292	↓
Bacteroidetes	Sphingobacteriaceae	*Pedobacter*	*P. kwangyangensis*	0.0076	− 0.0063	↓
Bacteroidetes	Sphingobacteriaceae	*Sphingobacterium*		0.0281	− 0.0100	↓
Bacteroidetes	Tannerellaceae	*Parabacteroides*		0.0152	− 0.0373	↓
Bacteroidetes	Tannerellaceae	*Parabacteroides*	*P. distasonis*	0.0411	− 0.0229	↓
Firmicutes				0.0212	0.1176	↑
Firmicutes	Acidaminococcaceae	*Acidaminococcus*	*A. intestini*	0.0004	0.0034	↑
Firmicutes	Eubacteriaceae	*Eubacterium*	*E. biforme*	0.0016	0.0026	↑
Firmicutes	Gemellaceae			0.0016	0.0065	↑
Firmicutes	Gemellaceae	*Gemella*		0.0008	0.0075	↑
Firmicutes	Lachnospiraceae			0.0343	0.0604	↑
Firmicutes	Odoribacteraceae			0.0186	− 0.0157	↓
Firmicutes	Paenibacillaceae			0.0156	0.0092	↑
Firmicutes	Ruminococcaceae	*Oscillospira*	*O. eae*	0.0211	− 0.0225	↓
Firmicutes	Ruminococcaceae	*Ruminococcus*	*R. gnavus*	0.0156	0.0163	↑
Firmicutes	Selenomonadaceae	*Megamonas*		0.0004	0.0023	↑
Firmicutes	Selenomonadaceae	*Megamonas*	*M. funiformis*	0.0004	0.0023	↑
Firmicutes	Selenomonadaceae	*Mitsuokella*		1.98 × 10^**−**8^	0.0036	↑
Firmicutes	Streptococcaceae			0.0075	0.0106	↑
Firmicutes	Streptococcaceae	*Streptococcus*		0.0082	0.0102	↑
Firmicutes	Thermicanaceae			0.0196	0.0067	↑
Firmicutes	Thermicanaceae	*Thermicanus*		0.0196	0.0067	↑
Firmicutes	Veillonellaceae	*Megasphaera*		1.01 × 10^**−**8^	0.0054	↑
Firmicutes	Veillonellaceae	*Megasphaera*	*M. hominis*	0.0001	0.0050	↑
Firmicutes	Veillonellaceae	*Veillonella*		0.0025	0.0088	↑
Firmicutes	Veillonellaceae	*Veillonella*	*V. atypica*	0.0410	0.0035	↑
Proteobacteria	Alcaligenaceae			0.0473	− 0.0213	↓
Proteobacteria	Desulfovibrionaceae	*Desulfovibrio*	*D. piger*	0.0111	0.0062	↑
Proteobacteria	Enterobacteriaceae	*Candidatus Blochmannia*	*C. B. rufipes*	3.49 × 10^**−**8^	− 0.0498	↓
Proteobacteria	Enterobacteriaceae	*Enterobacter*		0.0024	0.0146	↑
Proteobacteria	Enterobacteriaceae	*Escherichia*		0.0183	0.0221	↑
Proteobacteria	Enterobacteriaceae	*Escherichia*	*E. albertii*	0.0197	0.01954	↑
Proteobacteria	Enterobacteriaceae	*Klebsiella*		0.0006	0.01168	↑
Proteobacteria	Sutterellaceae	*Sutterella*		0.0462	− 0.0215	↓
Proteobacteria	Yersiniaceae	*Serratia*		0.0035	0.0114	↑

A multivariate association with linear models (MaAsLin) was used to perform a multivariate analysis on GM composition between OB and NW (adjusted for sex, age and smoking status) on the Galaxy Computational Tool v.3.5.2. NW = normal-weight healthy controls, OB = overweight and obese patients, *q*: *p* values adjusted for Benjamini and Hochberg false discovery rate (FDR) correction test for multiple comparisons (FDR < 0.05), Coefficient = median difference between OB and NW (median relative abundance in OB minus median relative abundance in NW), ↓ = significantly reduced in OB, ↑ = significantly increased in OB. *q* equal to or less than 0.05 was considered statistically significant.

**Figure 3:**
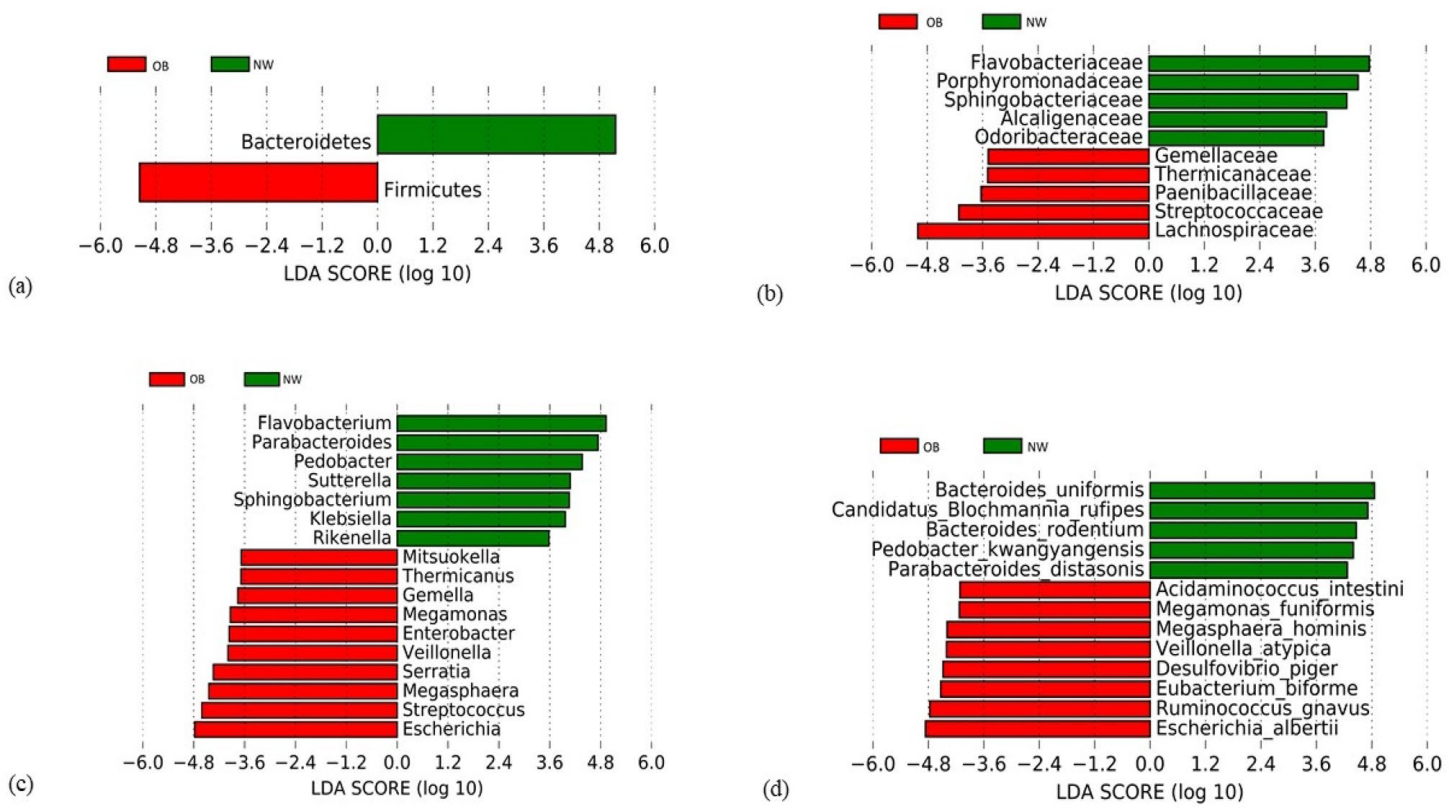
Linear Discriminant Analysis Effect Size (LEfSe) of microbial taxa between OB and NW. LEfSe plots of taxonomic biomarkers were generated on Galaxy computational tool v.1.0. (https://huttenhower.sph.harvard.edu/galaxy/). Results were ranked by their Linear Discriminant Analysis (LDA) score. Red bacterial taxa were more abundant in OB; green bacterial taxa were more abundant in NW. NW = normal-weight healthy controls, OB = overweight and obese patients; (**a**) Phylum level, (**b**) Family level, (**c**) Genus level, (**d**) Species level.

**Figure 4:**
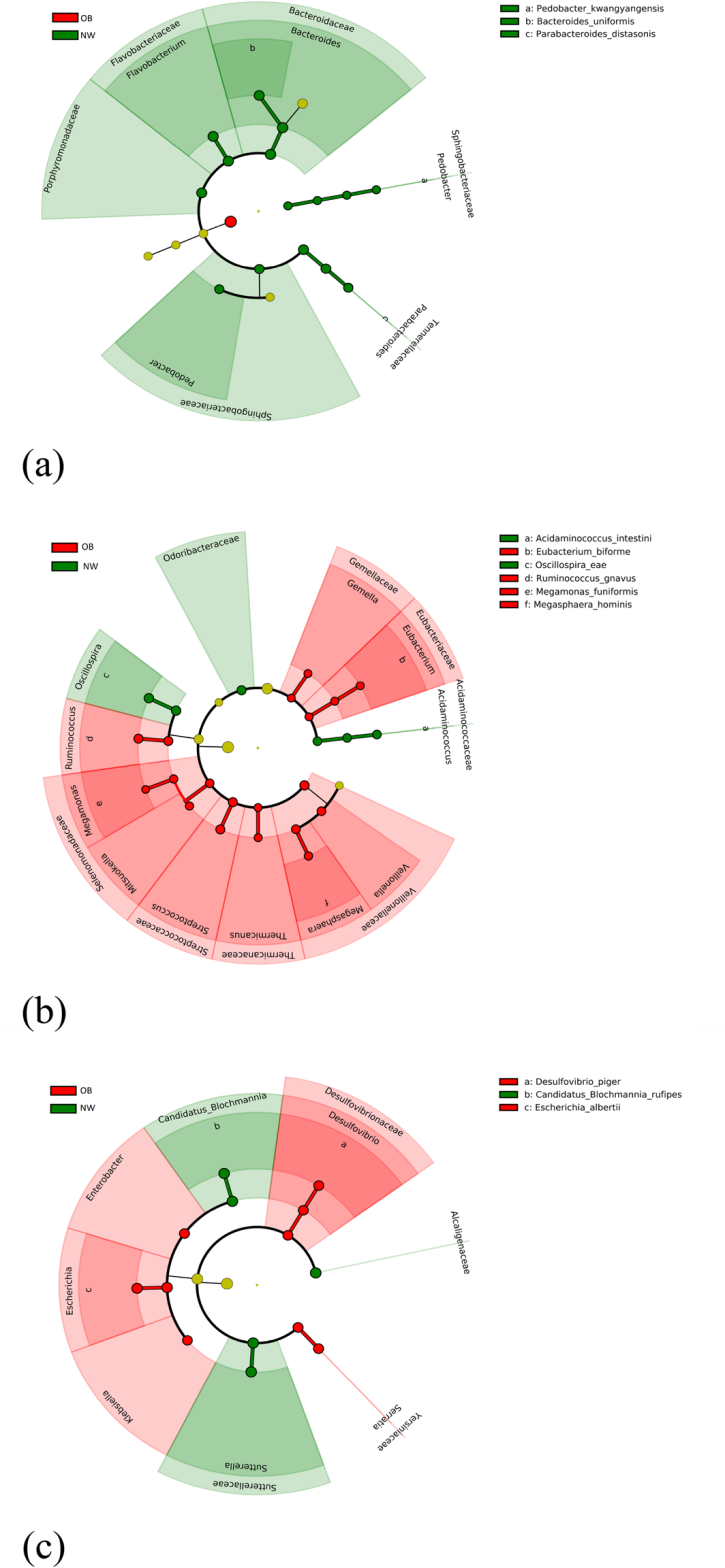
Linear Discriminant Analysis Effect Size (LEfSe) cladogram of microbial taxa between OB and NW. LEfSe algorithm was performed on Galaxy computational tool v.1.0. (https://huttenhower.sph.harvard.edu/galaxy/). The diameter of each circle is proportional to the abundance of the taxon. Only the differentially abundant taxa at the different taxonomic ranks were indicated. Red bacterial taxa were more abundant in OB; green bacterial taxa were more abundant in NW. NW = normal-weight healthy controls, OB = overweight and obese patients; (**a**) Bacteroidetes phylum, (**b**) Firmicutes phylum, (**c**) Proteobacteria phylum.

The Linear Discriminant Analysis Effect Size (LEfSe) was additionally performed on statistically significant bacterial taxa obtained by the multivariate analysis and confirmed after the False Discovery Rate (FDR) adjustment, in order to consider not only the statistical significance but also the biological consistency. Results were ranked by their Linear Discriminant Analysis (LDA) score (see Figs. 3, 4): the Bacteroidetes phylum and its members Flavobacteriaceae, *Flavobacterium,* and *Bacteroides* spp. were identified as the main biomarkers in NW, whereas in the OB group the strongest associations were related to Firmicutes phylum and its taxa Lachnospiraceae and *Megasphaera*, and to *Escherichia* and *E. albertii* (belonging to the Proteobacteria phylum).

The multivariate analysis on GM composition across different BMI categories showed that the major significant changes affect the group of patients with class III obesity, compared to NW. Specifically, a significant increase in the relative abundance of taxa belonging to the Firmicutes phylum (*Cateribacterium* and *Mitsuokella*), and a significant reduction in that of *Bacteroides uniformis* (Bacteroidetes phylum) and *Candidatus Blochmannia rufipes* (Proteobacteria phylum) was observed in patients (see Supplementary Table S10 and Fig. S3). Comparing the GM of the two subgroups of patients discordant for metabolic syndrome no statistically significant differences by the multivariate analysis were observed, but different taxa were significantly altered when comparing each subgroup of patients (OWMS and OMS) with NW (see Supplementary Tables S11 and S12 and Figs. S4 and S5 online).

### Spearman correlation between gut microbiota alterations and clinical variables in OB and NW

**Figure 5:**
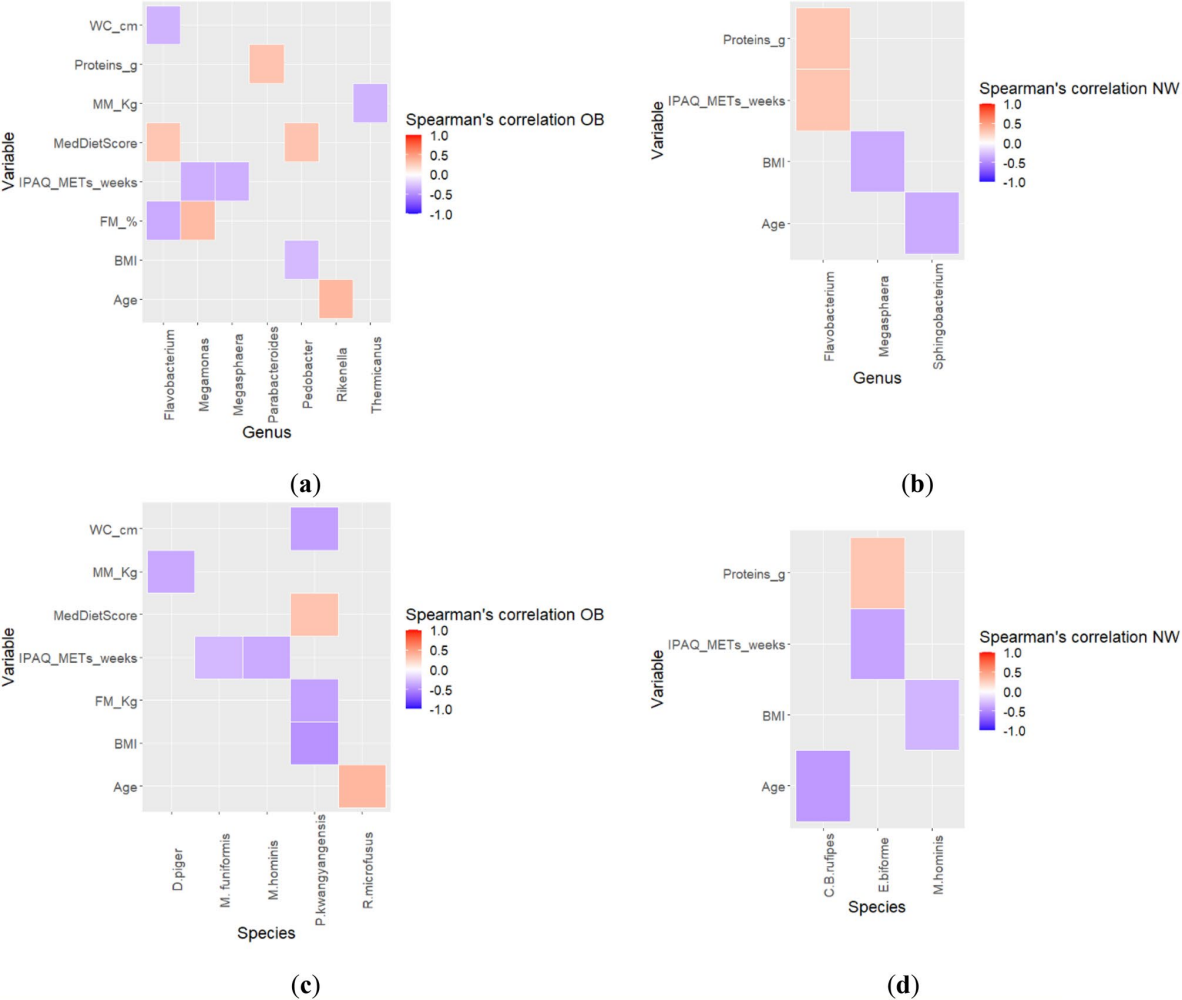
Spearman correlation analysis between GM alterations and clinical variables in OB and NW. Heatmaps were generated in R software v.3.5.2 (ggplot2 package). The correlation heatmap is used to represent significant statistical correlation values (Rho) between gut microbiota genera and species significant at the multivariate analysis and clinical features. In heatmap, red squares indicate significant positive correlations (Rho > 0.5, *p* < 0.05) and blue squares indicate significant negative correlations (Rho < − 0.5, *p* < 0.05). Only *p* < 0.05 are shown.

The taxa associated with OB were positively correlated with fat mass (*Megamonas*) and negatively correlated with the level of physical activity (*Megamonas*, *M. funiformis*, *Megasphaera*, *M. hominis*) and with the muscle mass (Thermicanaceae, *Thermicanus*, *D. piger*). On the other hand, the taxa less abundant in OB compared to NW were negatively correlated with fat mass (Flavobacteriaceae, *Flavobacterium* Porphyromonadaceae, *P. kwangyangensis*), waist circumference (Flavobacteriaceae, *Flavobacterium*, *P. kwangyangensis*) and BMI (Flavobacteriaceae, *Pedobacter*); instead, some taxa were positively correlated with BMI (Odoribacteraceae) and MedDietScore (Flavobacteriaceae, *Flavobacterium*, Sphingobacteriaceae, *Pedobacter*). The taxa associated to NW were negatively correlated with age (*Sphingobacterium*), while *Flavobacterium* positively correlated with daily protein intake and the level of physical activity. The taxa less abundant in NW were negatively correlated with the level of physical activity (*Eubacterium biforme*), BMI (*Megasphaera*, *M. hominis*) and age (*Blochmannia Candidatus rufipes*). Heatmaps of Spearman correlation analysis are shown in the Fig. 5, while the coefficient values and *p* values for each significant correlation can be found as Supplementary Tables S13 and S14.

### Functional metagenome prediction analysis

**Figure 6:**
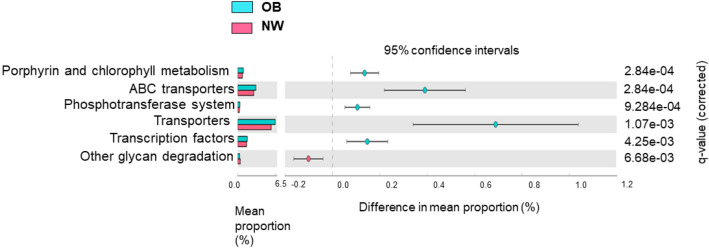
Statistically significant differences in predicted metabolic pathways between OB and NW. Phylogenetic Investigation of Communities by Reconstruction of Unobserved States (PICRUSt) algorithm was performed on Galaxy software v.1.0. (https://galaxy.morganlangille.com/) to infer metagenome composition in the samples by analyzing OTUs generated by QIIME pipeline. Bacterial metabolic pathways were predicted and classified by Kyoto Encyclopedia of Genes and Genomes (KEGG). Statistically differences were analyzed for all metabolism pathways using the Statistical Analysis of Metagenomic Profiles (STAMP) software. Pathways more abundant in OB are on the positive side (light-blue circle with 95% CI); pathways less abundant in OB are on the negative side (pink circle with 95% CI). The statistical significance was tested using Welch’s test, with a Storey False Discovery Rate (FDR) correction. *q* equal to or less than 0.05 was considered statistically significant. Mean proportions are shown in stacks (OB = light- blue; NW = pink). The difference in mean proportions indicates the mean proportion OB minus the mean proportion NW.

A comparative prediction analysis of the functional metagenome was performed using the phylogenetic survey of communities by unobserved state reconstruction (PICRUSt). A total of six different significantly metabolic pathways were identified by comparing OB and NW (see Fig. 6). In particular, a metabolic pathway related to the metabolism of cofactors and vitamins (porphyrin and chlorophyll metabolism), two pathways involved in membrane transport (ABC transporter, phosphotransferase system), transporter pathway and transcription pathway were most expressed in OB. Conversely, a metabolic pathway related to the metabolism of glycan biosynthesis (other glycan degradation) was enriched in NW.

## Discussion

In the present study, we characterized the distinctive features of the GM from overweight/obese patients (OB), compared to normal-weight controls (NW), both of Sardinian origin by using 16S rRNA gene sequencing on the MiSeq Illumina platform.

The alpha diversity in OB was lower than in NW, albeit no significant difference in the Shannon index was observed. This data partially agrees with previous studies, considering that the literature is not in line with a specific trend^17–20^; but it should be pointed out that the association between obesity and alpha diversity could be limited only to specific ethnic groups^21^, consistent with our findings.

The analysis of beta diversity showed a significant difference between OB and NW, partly in line with the literature^3,22^, highlighting the variation of microbial communities between study cohorts.

Linear discriminant analysis Effect Size (LEfSe) analysis showed microbial alterations in GM of OB group compared to control subjects. We observed that the Firmicutes/Bacteroidetes (F/B) ratio was significantly higher in OB, with a value more than twice that in NW. This data is in agreement with many other studies, while others did not report statistically significant differences, or even find an opposite relationship^23^. Increased F/B ratio in obese subjects is thought to lead to more efficient carbohydrate fermentation patterns and increased nutrient absorption^24–26^. Polysaccharides fermentation of dietary fibers by members of the Firmicutes phylum leads to the production of short-chain fatty acids (SCFAs) including acetate, propionate and butyrate, which are mostly found in the human colon^27^. Although SCFAs are known to have a beneficial role in food intake and body weight through binding to G protein-coupled receptors (GPR41 and GPR43), this process could be attenuated in obesity after high-carbohydrate diets, resulting in an increase in intestinal energy harvesting and hepatic lipogenesis^28^. Our results show the significant increase in the relative abundance of several bacterial taxa involved in the production of SCFAs in OB, mainly belonging to the Firmicutes phylum, including Lachnospiraceae and *Ruminococcus gnavus* (belonging to the Ruminococcaceae family). These are the predominant Firmicutes families of the human colon, which include the major butyrate-producing species, as well as species that produce butyrate or propionate from lactate and species that perform reductive acetogenesis^29^. Although Lachnospiraceae are beneficial bacteria, the increase in their abundance has been associated with metabolic diseases in both human and animal studies, probably due to the production of SCFAs different from butyrate^30^. In fact, emerging evidence indicates the pathological effect of specific SCFAs, such as acetate and propionate, in various disorders including obesity^31,32^. Consistently, our OB cohort showed a significant increase in the relative abundance of *Acidaminococcus intestini*, *Eubacterium biforme*, Odoribacteraceae, *Mitsuokella*, *Thermicanus*, *Veillonella* and *Veillonella atypica*, all belonging to the Firmicutes phylum and, except *Eubacterium biforme* (butyrate producer), able to produce different SCFAs from butyrate, such as acetate and propionate^33–35^. As for butyrate, it can be produced from glutamate and lysine amino acids by some bacteria, including *Megasphaera*, in a pathway that involves the production of ammonia, which can have harmful effects^36^. Noteworthy, it has been hypothesized that a reduced intake of non-digestible dietary carbohydrates in obese patients could lead to increased production of SCFAs through increased microbial conversion of amino acids (AA) into SCFAs^37^. In our OB cohort, the relative abundance of different bacterial taxa capable of fermenting amino acids, such as *Eubacterium biforme*, *Veillonella*, *Veillonella atypica* (all belonging to the Firmicutes phylum), *Escherichia* and *Escherichia albertii* (belonging to Proteobacteria taxa), was significantly higher than in NW.

Within Firmicutes phylum, the relative abundance of *R. gnavus* in OB was a significantly increased. It has been shown that *R. gnavus* has the ability to degrade mucin and use mucin-derived glycans as nutrients, as well as to produce propanol and propionate as metabolic end products^38^. The increase in the relative abundance of *R. gnavus* in the OB cohort could be a consequence of the significant reduced intake of non-starch dietary glycans, and of the excess of lipid dietary intake and low fibers intake in the same subjects. In fact, HFD, paired with the reduction of dietary fibers, could favor the growth of bacterial species capable of digesting host mucosal glycans^39^. The ability of *R. gnavus* to utilize mucin-derived glycans may favor its early colonization in the gut and ensure endogenous glycans to other non-mucin-degrading intestinal bacteria. Interestingly, the glycans of the host mucin are the most important source of sulfate in the intestine and some bacteria, such as *Desulfovibrio piger* (whose relative abundance is significantly increased in our OB cohort), are able to remove by-products of gaseous fermentation (such as hydrogen) by reducing sulfate, resulting in production of hydrogen sulfide. Moreover, hydrogen sulfide can further facilitate the degradation of mucin by acting on the disulfide bonds^40^. The hydrogen sulfide promotes both toxic (for intestinal epithelial cells) and pro-inflammatory effects, and its concentration, as well as that of producing bacteria, is higher in inflammatory bowel diseases (IBD)^40^. In addition, *R. gnavus* can induce the secretion of inflammatory cytokines through the synthesis and secretion of a complex glucorhamnan polysaccharide^41^. Therefore, a synergistic metabolic activity between *R. gnavus* and *D. piger*, as a cause of the intestinal barrier damage, with consequent exacerbation of the inflammatory process, could be hypothesized.

Regarding other taxa within the Firmicutes phylum, the relative abundance of *Oscillospira eae* in the OB group was significantly reduced. Previous evidence indicated that *Oscillospira* was enriched in healthy-weight subjects; moreover, it has been associated to low BMI and it has also been thought to promote human leanness^42^.

The significant reduction in the relative abundance of Bacteroidetes phylum in our OB group compared to NW is another possible diet-induced effect. In fact, Bacteroidetes encode a greater number of carbohydrate-degrading enzymes than Firmicutes^43^. Consistently, the relative abundance of *Bacteroides uniformis* was significantly reduced in the same subjects. *B. uniformis*, one of the predominant species in the human colon, is able to degrade a heterogeneous number of polysaccharides^43^. In the present study a significant increase in the relative abundance of Porphyromonadaceae, Sphingobacteriaceae, Flavobacteriaceae (and related members *Sphingobacterium* and *Flavobacterium*) was found in NW group. Previous studies on animal models have shown that the abundance of these families can be influenced by lifestyle factors, with a positive modulation exerted by physical exercise and a negative modulation exerted by antibiotics and high-fat diet^44,45^. A significant decrease in the relative abundance of the genera *Rikenella*, *Pedobacter*, *Parabacteroides* and *R. microfusus*, *P. kwangyangensis*, *P. distasonis* species was also observed in OB. Del Chierico et al. have recently associated both *Parabacteroides* and *Parabacteroides distasonis* with NW, in agreement with our study^14^.

In addition, OB presented a significant increase in the relative abundance of different bacterial taxa belonging to the Proteobacteria phylum, as *Desulfovibrio piger* and to the Enterobacteriaceae family, such as *Enterobacter*, *Escherichia*, *Escherichia albertii*, *Klebsiella* and *Serratia* (in line with other human studies^46^). The relative abundance of Alcaligenaceae, *Sutterella* and *Candidatus Blochmannia rufipes* was, on the other hand, significantly decreased. *Escherichia* and *Escherichia albertii* were also identified among the main biomarkers of obesity in our study. These bacteria represent an important source of LPS, which can affect the intestinal permeability leading to an increased concentration of LPS in plasma (endotoxemia), connected with the chronic low-grade inflammation typical of obese subjects^46^. Regarding the other taxa previously mentioned, the possible implication of the Alcaligenaceae family in the intestinal reabsorption of cholesterol has been suggested^47^. On the other hand, *Sutterella* has been reported to have beneficial effects on glucose metabolism in T2D patients after gastric bypass Roux-en-Y (RYGB)^48^; however, the potential pathophysiological role of *Candidatus Blochmannia rufipes* in humans is currently unknown.

In the present work, some of the identified microbial markers were correlated with clinical parameters such as body composition, physical activity levels, extent of adherence to MD and age. The positive correlation between Firmicutes taxa and the percentage of body fat is not surprising, considering that an increase in Firmicutes can be associated with augmented hepatic de novo lipogenesis, increased uptake of fatty acids and storage of triglycerides in adipocytes, suppression of skeletal muscle fatty acid oxidation, decrease in the gut motility, and consequent increase of intestinal transit time and nutrients absorption rate^49,50^. Regarding the negative association between Firmicutes and the muscle mass, recent findings on animal models showed that gut dysbiosis may increase inflammatory markers and reactive oxygen species generation, all contributing to skeletal muscle atrophy^51^. Similarly, findings from animal models have suggested an inverse association between Firmicutes and physical exercise, especially at high intensity^52^. In contrast, we observed a negative correlation between Bacteroidetes and the percentage of body fat. The mechanisms linking the capacity of Bacteroidetes members to influence body weight remain to be ascertained, but a modulation via secondary bile acid-activated Farnesoid X Receptor (FXR) signaling in the liver, and via succinate-activated intestinal gluconeogenesis has been recently suggested in an animal model^53^. At the same time, the administration of *Bacteroides uniformis*, depleted in our OB cohort, improved metabolic and immune dysfunction associated with intestinal dysbiosis in obese mice^4^.

Several bacterial taxa positively correlated with the MedDietScore, such as Flavobacteriaceae, Sphingobacteriaceae, *Flavobacterium*, *Pedobacter* and *P. kwangyangensis*. It is well known that the MD plays a protective role in the prevention of various diseases, including obesity. Furthermore, recent studies have suggested that both polyphenols and fibers in MD are able to positively modify intestinal microbiota^54^. In a previous study, in mice with HFD-induced non-alcoholic fatty liver disease, *Flavobacterium* significantly increased in terms of relative abundance following supplementation of quercetin, a polyphenolic compound^55^. In our study, *Flavobacterium* and *E. biforme* were positively and negatively correlated, respectively, to physical activity level in NW. In line with our findings and as stated in a study by Carbajo-Pescador et al., exercise restored HFD-induced microbial imbalance in rats, including the relative abundance of *Flavobacterium*^56^. At the same time, a significant reduction in species belonging to the genus *Eubacterium*, including *E. biforme*, was observed after sustained physical activity in humans^57^.

Lastly, we performed a comparative prediction analysis of the functional metagenome in order to identify the microbial metabolic pathways that underwent significant alterations in OB. Distinct features of the microbial metabolic profile between OB and NW were observed. More specifically, an increase in transcription, membrane transport and cofactors of vitamins metabolism pathways was observed in OB patients. Regarding the latter, we documented a significant increase in porphyrin and chlorophyll metabolism in OB compared to NW. This pathway has been previously associated with intestinal dysbiosis^58^ and shown to be overexpressed after an HFD in animal models. However, the connection with obesity remains to be ascertained. In addition, phosphotransferase systems from the related pathway “membrane transport” were expressed more in obese patients, in agreement with previous studies^8,59–61^. These transporters are involved in carbohydrates breakdown and phosphorylation, utilization of nitrogen and phosphorus and virulence of some pathogens. In an animal model, the high-fat/high-sugar Western diet was associated with an enrichment of the phosphotransferase system, suggesting that this pathway can be modulated by diet^61^. In line with other studies, a depletion in glycan metabolism was also observed in OB^14,60^. Interestingly, Firmicutes encode fewer carbohydrate-degrading enzymes than Bacteroidetes but possess a greater number of ABC carbohydrate transporters^39^. This evidence is in line with our results, which highlighted the significant increase of ABC transporters in OB that are involved in the transport of a variety of substrates, including nutrients, toxins, antibiotics, and xenobiotics^62^. Noteworthy, genes encoding ABC transporters specific for glycans are often located adjacent to those encoding glycoside hydrolases (with which they are co-expressed) in Firmicutes but not in Bacteroidetes. This is probably a glycan acquisition strategy that Firmicutes have developed to make the use of glycans more efficient^39^. In line with the present study, Hou et al. observed an association between the increase of membrane transport (ABC transporters) and obesity^60^.

A potential limitation of our study was the prevalence of women in the analyzed cohorts. It was difficult to recruit a homogeneous number of subjects of both sexes due to the lower propensity of obese men to participate in weight- loss programs, as previously reported^63^. To preclude that any significant GM changes reflecting gender-driven selection, we established two study cohorts (pathological and control subjects) matched for sex, as confirmed by the statistically not significant values ​​obtained by Pearson's chi-squared test. Moreover, MaAsLin was used to perform multivariate analysis on GM composition between OB and NW, adjusted for sex, to exclude the effect of gender as a confounding factor. Therefore, our next step will be to expand the study cohorts with an equal number of males and females, in order to confirm that findings could be considered generalized to obese subjects.

The present study confirmed gut dysbiosis in OB patients, extending our knowledge of the association between GM and obesity in Italy. Our findings are only partially in line with those of previous Italian works, probably due also to the influence exerted by the region of origin on the microbiota profile, which could make the latter partially distinctive of obese Sardinians^64^. Our study population was characterized by an excessive content in SCFAs producing and pro-inflammatory bacteria, which can be identified as targets for therapeutic approaches.

## Methods

### Ethics declarations

Informed written consents were obtained from all participants before participation in the study. Institutional review boards and human subject committees at participating institutions approved the study (Comitato Etico Indipendente della A.O.U. di Cagliari, C.E.I., Prot.PG/2020/2973). The study was conducted in accordance with the Declaration of Helsinki.

### Study design and characteristics of subjects

A cross-sectional analytic study evaluating the distinctive signatures in gut microbiota (GM) of overweight/obese patients (OB) was performed. Overall, 92 subjects of Sardinian origin, of which 46 healthy normal-weight controls (NW) and 46 OB were recruited. The latter included 30 obese/overweight patients with metabolic syndrome (OMS) and 16 obese/overweight patients without metabolic syndrome (OWMS). Cases and controls of both sexes and aged ≥ 18 years, matched for sex, age, and smoking status, were included in the study by the Obesity Center of the University Hospital of Cagliari (Sardinia, Italy) and by the Department of Biomedical Sciences of the same Hospital University. The inclusion criteria for the OB group were a BMI ≥ 25 kg/m^2^ and a not specific diet within the 12 months before the recruitment (diet-free condition), in order to characterize the GM composition of patients during their usual dietary habits. The inclusion criteria for NW were a BMI between 18.5 and 24.9 kg/m^2^, the absence of any gastrointestinal or metabolic disorder, a diet-free requirement and no changes in body weight in the last 2 years. For both OB and NW groups, exclusion criteria included the presence of Intestinal Bowel Disease (IBD), use of antibiotics or proton pump inhibitors, prebiotics, probiotics or dietary supplements within 3 months prior to the sample collection, history of cancer, presence of neurological or psychiatric illness.

Clinical data from each study participant, including demographic and anthropometric data, lifestyle factors (smoking status, alcohol consumption, nutritional data and level of physical activity) and the presence of comorbidities such as dyslipidemia, glucose metabolism alterations and hypertension, were collected (Table 1). All clinical evaluations were performed contextually to the sampling date. Patients with or without metabolic syndrome diagnosis were further stratified into the OBMS and OWMS groups, respectively (see Supplementary Table S1 online). MS was diagnosed according to the International Diabetes Federation (IDF) criteria^65^ (see Supplementary Materials “Diagnosis of metabolic syndrome”). Anthropometric and lifestyle factor assessments can also be found as Supplementary Materials in the sections "Anthropometric evaluation" and "Lifestyle factor assessment".

### Sample collection

Stool samples from each subject were collected at outpatient facilities and delivered to the laboratory within 3 h. Fresh samples were stored at − 80 °C until further processing.

### Genomic DNA extraction from fecal sample and quantification of bacterial DNA

DNA extraction from fecal samples was performed using the QIAamp DNA stool MiniKit according to the instructions of the manufacturer (Qiagen), with minor modifications. Quantitative PCR (qPCR) was performed using degenerate primers encompassing the V3 and V4 hypervariable region of the bacterial 16S rRNA gene, as previously described^66^.

### 16S libraries preparation and sequencing

The protocol of library preparation and sequencing has been described in detail elsewhere^66^. 16S barcoded amplicon libraries were generated using primers targeting the V3-V4 hypervariable region of the bacterial 16S rRNA gene and the Nextera XT index kit (Illumina, inc.), and their size and quality were verified using Agilent DNA 1000 Analysis kit (Agilent Technologies) on the Agilent 2100 Bioanalyzer system (Agilent Technologies). Genomic libraries were quantified on the Qubit 3.0 Fluorometer by using the Qubit dsDNA HS (High Sensitivity) Assay Kit, according to the instructions of the manufacturer (Thermo Fisher Scientific), normalized to a concentration equal to 4 nM, then pooled. Pooled library and the adapter-ligated library PhiX v3 used as a control were denaturated and diluited to equal concentration (8 pM) and subsequently combined in order to obtain a PhiX concentration equal to 5% of the total volume. Combined 16S library and PhiX control were further denatured and, finally, sequenced on the MiSeq platform using MiSeq v3 Reagent Kit (Illumina).

### Data and statistical analysis

Analysis of the data generated on the Miseq System was carried out using the BaseSpace 16S Metagenomics App (Illumina), whereas operational taxonomic unit (OTU) mapping to the Greengenes database (v.13.8) were performed using the Quantitative Insights Into Microbial Ecology (QIIME) platform (v.1.8.0), as previous described^66^.

Alpha diversity was assessed with the script alpha rarefaction.py in QIIME in order to obtain the Shannon index. Alpha diversities and Firmicutes/Bacteroidetes ratio were analyzed by using the Kruskal–Wallis test followed by Bonferroni correction for multiple comparisons. Beta diversity was generated in R-vegan, using Bray–Curtis distance. Principal Coordinates Analysis (PCoA) based on Bray–Curtis distance matrix was conducted in vegan. The statistical significance of beta diversity among the two groups was determined with Permutational Multivariate Analysis of Variance (PERMANOVA) (R-vegan, function adonis). Overall *p* value obtained from multiple comparisons was confirmed through pairwise PERMANOVA test. The analysis of the taxonomic levels was performed in R software v.3.5.2. Only bacteria present in at least 25% of our samples and with a relative abundance ≥ 0.1% in cases and/or controls were considered. A multivariate association with linear models (MaAsLin) was used to perform a multivariate analysis, adjusted for sex, age and smoking status; *p* values were adjusted for Benjamini and Hochberg false discovery rate (FDR) correction test for multiple comparisons (FDR < 0.05). In addition, Linear discriminant analysis Effect Size (LEfSe) was employed for the identification of biomarkers. The LEfSe algorithm and the MaAsLin algorithm were performed on the Galaxy computational tool (http://huttenhower.sph.harvard.edu/galaxy/). The association between the relative abundance of significant taxonomic levels and clinical parameters was evaluated by calculating the Spearman’s correlation.

The differences in metabolic pathways between OB and NW were analyzed in the Statistical Analysis of Metagenomic Profiles (STAMP) software and R software. The statistical significance was tested using Welch’s test, with a Storey FDR correction. All the *p* values were adjusted for FDR and *q*-values < 0.05 were considered as statistically significant.

## Supplementary Information

Supplementary information.

## Data Availability

Our sequence data for the 16S rRNA gene was deposited in the European Nucleotide Archive (ENA) (https://www.ebi.ac.uk/ena), under the study accession number PRJEB39037 (http://www.ebi.ac.uk/ena/data/view/PRJEB39037).

## Abbreviations

GM: Gut microbiota
NW: Normal-weight healthy controls
OB: Overweight and obese patients
OMS: Overweight and obese patients with metabolic syndrome
OWMS: Overweight and obese patients without metabolic syndrome
SCFAs: Short chain fatty acids
