# Supplementary material for: Gut microbiota markers associated with obesity and overweight in Italian adults
Source: Sci Rep. 2021 Mar 9;11:5532. doi: 10.1038/s41598-021-84928-w (PMC7943584; doi:10.1038/s41598-021-84928-w)
Supplement: Supplementary file 1 — Supplementary information. [file 41598_2021_84928_MOESM1_ESM.pdf]

Gut microbiota markers associated with obesity and overweight in Italian adults

Vanessa Palmas[1†], Silvia Pisanu[1†], Veronica Madau[1], Emanuela Casula[1], Andrea Deledda[2], Roberto Cusano[3], Paolo Uva[3], Sarah Vascellari[1], Andrea Loviselli[2], Aldo Manzin[1*], Fernanda Velluzzi[2*]

[1]Department of Biomedical Sciences, University of Cagliari, Cagliari, Italy
[2]Department of Medical Sciences and Public Health, University of Cagliari, Cagliari, Italy
[3]CRS4, Science and Technology Park Polaris, Piscina Manna, Pula, Cagliari, Italy

[†]These authors contributed equally to the work
*These authors contributed equally to the work

Correspondence to: Prof. Aldo Manzin, Department of Biomedical Sciences, University of Cagliari, Microbiology and Virology Unit, Azienda Ospedaliero-Universitaria Cagliari, Strada Statale 554- bivio Sestu, I-09042 Monserrato, Cagliari (email: aldo.manzin@gmail.com).

Methods

Diagnosis of metabolic syndrome

Metabolic syndrome was diagnosed according to the International Diabetes Federation (IDF) criteria [1], which include the presence of central obesity (defined as waist circumference (WC) ≥ 94cm for men and ≥ 80cm for women) plus any two of the following four cardiometabolic risk factors: raised blood pressure (systolic ≥ 130 mmHg and / or diastolic ≥ 85 mmHg or treatment of previously diagnosed hypertension), triglycerides (TG) ≥ 150 mg/dl, low plasma levels of high-density lipoprotein (HDL) cholesterol (<40 mg/dl in males and < 50 mg/dl in females or specific treatment for these lipid abnormalities), impaired fasting glycemia (IFG): fasting plasma glucose (FPG) ≥ 100 mg/dl, or previously diagnosed type 2 diabetes (T2DM).

Anthropometric evaluation

Anthropometric evaluation including height, weight, waist circumference measurement and impedance analysis was conducted at room temperature and under fasting conditions. The height expressed in cm was measured with a stadiometer, after removal of the shoes, with the participant's head being in the "Frankfort plane". The body weight, expressed in kilograms, was measured with an impedance scale (TANITA BC-420), also used for the analysis of the body composition. Waist circumference, expressed in centimeters, was measured using recommendations of the Airlie (VA) Consensus Conference. The impedance analysis was performed at room temperature, under fasting condition for at least 2 hours, and with the last moderate-intense physical activities at least 24 hours previously. BMI was calculated by the ratio of weight in kilograms and height in meters squared: normal range (normal-weight condition) was considered 18.5-24.99 kg/m2, the range for overweight was 25-29.99 kg/m2, for obese class I was 30-34.99 kg/m2, for obese class II was 35-39.99 kg/m2 and for obese class III greater than or equal to 40 kg/m2.

Lifestyle factors assessment

A weighted 3-day food intake record (3d-FR, including 2 days of the week and 1 weekend day) was analyzed with the Winfood ® software (v.3.0) and the following parameters were obtained: average daily caloric intake expressed in kcal, daily percentage of macronutrients intake (carbohydrates, lipids, and proteins), daily percentage of saturated lipids intake (on the total lipids intake), and daily intake of fiber in grams.

Overall, correct eating habits, based on the analysis of food diaries, were assessed on the extent of adherence to the Mediterranean diet (MD), attested by the calculation of the Mediterranean Diet Score (MedDietScore, range 0-55), a composite index of eleven food items, whose higher score indicate higher compliance [2].

The extent of alcohol consumption was established according to the MD model, defining "moderate" as a consumption of alcohol lower than 300 mL/ day, "rare" as the occasional consumption of alcohol (maximum 1 time per week) and "none" as the absence of alcohol consumption.

The level of physical activity was assessed by using the International Physical Activity Questionnaire short-form (IPAQ-SF), which consists of seven questions to capture the average daily time spent sitting, walking, and engaging in moderate and vigorous physical activity over the last seven days [3].

Table S1. Clinical characteristics of metabolic syndrome discordant obese patient subgroups

	NW	OWMS	OMS	*p* OWMS *vs* NW	*p* OMS *vs* NW	*p* OMS vs OWMS
N	46	16	30			
Age (M ± SD)	49 ±11	45.3 ± 13.2	52.3 ± 11.2	0.259	0.229	0.066
Female (n, %)	40, 87.0	15, 93.8	25, 83.3	0.460	0.661	0.318
Anthropometric data						
BMI (M ± SD)	21.6 ± 2.1	34.9 ± 5.0	36.7 ± 6.5	**6.84 x 10^{-22}**	**1.91 x 10^{-23}**	0.338
Fat mass (kg)		36.0 ± 9.4	40.8 ± 12.9			0.201
Fat mass (%)		42.4 ±5.0	42.3 ± 6.1			0.985
Muscle mass (Kg)		45.4 ± 4.1	50.1 ± 13.4			0.182
Waist circumference (cm) (M ± SD)	73.7 ± 5.7	106.9 ± 16.4	113.2 ± 14.6	**1.69 x 10^{-17}**	**1.40 x 10^{-26}**	0.188
Overweight (N, %)		2, 12.5	3, 10.0			0.795
Obesity Class I (N; %)		7, 43.8	10, 33.3			0.486
Obesity Class II (N; %)		6, 37.5	11, 36.7			0.956
Obesity Class III (N, %)		1, 6.3	6, 20.0			0.216
Lifestyle factors						
Smoking status Yes (n, %)	8, 17.8	6, 37.5	6, 20.0	0.107	0.809	0.198
Alcohol consumption None (n, %)	10, 21.7	8, 50.0	13, 43.3	**0.032**	**0.045**	0.665
Alcohol consumption Rare (n, %)	24, 52.2	7, 43.8	10, 33.3	0.562	0.106	0.486
Alcohol consumption Moderate (n, %)	12, 26.1	1, 6.3	7, 23.3	0.093	0.786	0.145
Daily caloric intake kcal (M ± SD)	1467.8 ±162.3	1748.1 ± 556.3	1844.5 ± 669.0	**0.003**	**4.67 x 10^{-4}**	0.338
Daily carbohydrates intake % (M ± SD)	50.9 ± 3	49.8 ± 6.4	49.9 ± 6.7	0.323	0.379	0.930
Daily lipids intake % (M± SD)	27.2 ± 4.3	33.9 ± 6.7	32.8 ± 6.3	**2.24 10^{-5}**	**2.54 10^{-5}**	0.560
Daily saturated lipids intake/total lipids intake % (M± SD)	27.8 ± 4.3	36.0 ± 7.6	39.8 ± 6.0	**2.46 10^{-6}**	**1.74 10^{-14}**	0.091
Daily proteins intake grams (M± SD)	62.3 ± 8.8	69.1 ± 18.8	83.8 ± 52.4	0.058	**0.008**	0.288
Daily fiber intake grams (M± SD)	20.0 ± 3.2	16.8 ± 11.6	13.5 ± 4.3	0.088	**2.81 x 10^{-10}**	0.193
MedDietScore (M± SD)	33.4 ± 4.1	27.3 ± 6.1	28.8 ± 5.1	**2.47 x 10^{-5}**	**4.13 x 10^{-5}**	0.366
IPAQ METs/week (M ± SD)	2604.8 ± 135.4	652.2 ± 586.7	667.0 ± 626.7	**1.17 x 10^{-29}**	**5.35 x 10^{-32}**	0.938
Dyslipidemia						
Yes (n, %)	0	3, 18.8	25, 83.3			**1.91 x 10^{-5}**
Alteration in glucose metabolism*						
Yes (n, %)	0	8, 50.0	24, 80.0	NA		**0.035**
Hypertension						
Yes, %	0	4, 75.0	23, 76.7			**0.001**

*impaired Fasting Plasma Glucose (FPG) between 100–125 mg/dl (6.9 mmol/l), impaired glucose tolerance (IGT) if 2 h post-OGTT plasma glucose was 140–199 mg/dl (7.8-11.0 mmol/l), T2DM if FPG was ≥ 126 mg/dl (≥ 7 mmol/l) on two days apart, or if 2h post-OGTT plasma glucose was ≥ 200 mg/dl (≥ 11.1 mmol/l). The statistical significance was evaluated by *t* test for independent samples for continuous variables and by Pearson's chi-squared test for categorical variables. Bold values denote statistical significance ($p < 0.05$). NW= healthy normal-weight controls, OWMS= obese and overweight patients without metabolic syndrome, OMS= obese and overweight patients with metabolic syndrome.

Table S2. Alpha diversity analysis between OB vs NW

Shannon index	Median (range)	*p*
		0.840
**OB**	2.40 (1.17, 3.10)	
**NW**	2.39 (1.84, 2.68)	

Differences in the medians of Shannon index between OB and NW, evaluated by Kruskal-Wallis test. NW= normal-weight healthy controls, OB= overweight and obese patients. *p*-value equal to or less than 0.05 was considered statistically significant.

Table S3. Alpha diversity analysis in OB across different BMI categories

Shannon index		Median (range)	*p*
			0.780
NW		2.39 (1.84, 2.68)	
Overweight		2.17 (1.99, 2.54)	
OB	Class I	2.42 (1.79, 2.92)	
	Class II	2.42 (1.17, 3.10)	
	Class III	2.47 (1.54, 2.65)	

Differences in the medians of Shannon index in OB across different BMI categories, evaluated by Kruskal-Wallis test. NW= normal-weight healthy controls, OB= obese patients. *p*-value equal to or less than 0.05 was considered statistically significant.

Table S4. Alpha diversity analysis between NW, OWMS and OMS

Shannon index	Median (range)	*p*
		0.873
**NW**	2.36 (1.84, 2.68)	
**OWMS**	2.42 (1.94, 2.93)	
**OMS**	2.39 (1.17, 3.10)	

Differences in the medians of Shannon index between metabolic syndrome discordant obese patient subgroups, evaluated by Kruskal-Wallis test. NW= normal-weight healthy controls, OWMS= overweight and obese patients without metabolic syndrome, OMS= overweight and obese patients with metabolic syndrome. *p*-value equal to or less than 0.05 was considered statistically significant.

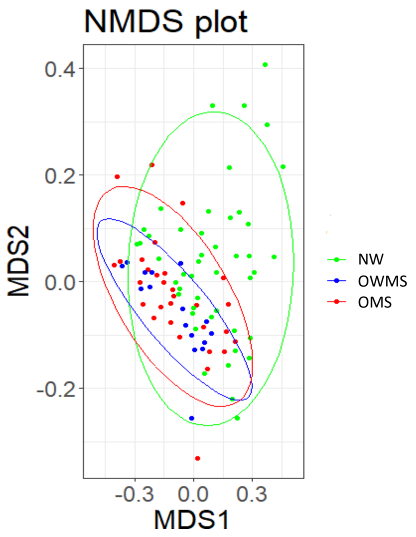

Fig. S1. GM beta diversity analysis between NW, OWMS and OMS

The three-dimensional scatter plots, generated using the Non-Metric Multidimensional Scaling (NMDS) based on Bray-Curtis distance in R software v.3.5.2 (ggplot2 package), showed a marked separation between the GM communities of groups. The statistical significance among the two groups was determined with Permutational Multivariate Analysis of Variance (PERMANOVA) (R-vegan, function adonis), adjusted for sex, age and smoking status (sum of squares= 0.642, mean of squares= 0.321, F= 2.955, p= 0.007). p- value equal to or less than 0.05 was considered statistically significant. Pairwise PERMANOVA test confirmed significant segregation only in the comparison between OMS and NW (p= 0.030).

Table S5. Median and range of the relative abundance of each taxonomic level in OB and NW

	OB		NW	
**Taxonomic profile**	**Median**	**Range (min, max)**	**Median**	**Range (min, max)**
Actinobacteria	2.12	(0.51, 22.23)	1.86	(0.14, 14.45)
Bacteroidetes	29.69	(2.94, 74.72)	40.59	(7.18, 83.43)
Chloroflexi	0.05	(0.02, 0.59)	0.08	(0.01, 0.67)
Cyanobacteria	0.16	(0.08, 1.97)	0.40	(0.05, 3.95)
Firmicutes	54.00	(15.39, 87.86)	47.10	(13.85, 67.75)
Proteobacteria	3.61	(1.36, 23.48)	3.18	(1.31, 30.61)
Synergistetes	0.06	(0.01, 0.25)	0.03	(0.00, 2.33)
Verrucomicrobia	0.13	(0.01, 36.7)	0.24	(0.00, 25.08)
Alcaligenaceae	0.13	(0.01, 6.49)	0.39	(0.00, 2.62)
Anaerobrancaceae	0.03	(0.00, 0.96)	0.06	(0.00, 2.17)
Bacteroidaceae	16.13	(1.05, 57.72)	25.53	(4.52, 66.51)
Bifidobacteriaceae	1.30	(0.09, 20.90)	1.05	(0.01, 13.86)
Caldilineaceae	0.05	(0.00, 0.58)	0.08	(0.01, 0.65)
Clostridiaceae	4.85	(1.47, 11.65)	4.55	(0.74, 20.62)
Comamonadaceae	0.03	(0.00, 0.93)	0.04	(0.00, 0.71)
Coprobacillaceae	0.05	(0.00, 1.61)	0.02	(0.00, 4.70)
Coriobacteriaceae	0.47	(0.06, 8.94)	0.22	(0.02, 3.52)
Desulfohalobiaceae	0.05	(0.00, 0.63)	0.07	(0.00, 1.27)
Desulfovibrionaceae	0.16	(0.01, 9.93)	0.27	(0.00, 2.61)
Enterobacteriaceae	0.71	(0.04, 21.37)	0.89	(0.03, 12.87)

Table S5. Median and range of the relative abundance of each taxonomic level in OB and NW

	OB		NW	
**Taxonomic profile**	**Median**	**Range (min, max)**	**Median**	**Range (min, max)**
Erysipelotrichaceae	0.21	(0.02, 3.85)	0.25	(0.01, 7.40)
Eubacteriaceae	0.09	(0.02, 0.36)	0.07	(0.02, 0.33)
Flavobacteriaceae	1.61	(0.04, 7.65)	3.89	(0.18, 21.96)
Gemellaceae	0.08	(0.02, 0.48)	0.05	(0.01, 0.17)
Lachnospiraceae	15.14	(3.35, 79.95)	10.39	(0.00, 26.07)
Lactobacillaceae	0.11	(0.01, 4.06)	0.08	(0.00, 1.09)
Microbacteriaceae	0.10	(0.04, 4.52)	0.10	(0.00, 6.01)
Nostocaceae	0.08	(0.03, 1.79)	0.13	(0.02, 3.61)
Odoribacteraceae	0.12	(0.00, 0.68)	0.28	(0.00, 3.18)
Paenibacillaceae	0.05	(0.01, 0.43)	0.03	(0.01, 0.36)
Paraprevotellaceae	0.08	(0.00, 14.47)	0.05	(0.00, 3.03)
Pasteurellaceae	0.02	(0.00, 2.19)	0.02	(0.00, 1.86)
Peptococcaceae	0.17	(0.04, 0.95)	0.18	(0.02, 1.57)
Porphyromonadaceae	1.16	(0.08, 6.86)	2.61	(0.09, 14.87)
Prevotellaceae	0.16	(0.01, 24.45)	0.04	(0.00, 39.80)
Rikenellaceae	0.02	(0.00, 4.27)	0.00	(0.00, 0.50)
Ruminococcaceae	15.24	(1.33, 52.40)	16.75	(4.23, 48.19)
Sphingobacteriaceae	0.38	(0.02, 3.45)	1.12	(0.15, 9.52)
Streptococcaceae	0.25	(0.06, 10.21)	0.10	(0.02. 1.48)
Streptomycetaceae	0.09	(0.04, 1.31)	0.07	(0.02, 0.86)
Succinivibrionaceae	0.00	(0.00, 0.26)	0.00	(0.00, 23.55)
Thermicanaceae	0.03	(0.00, 3.45)	0.01	(0.00, 0.32)
Thiotrichaceae	0.03	(0.00, 0,31)	0.06	(0.01, 0.78)
Veillonellaceae	4.23	(0.27, 38.39)	2.61	(0.08, 15.71)
Verrucomicrobiaceae	0.12	(0.00, 36.50)	0.24	(0.00, 25.00)
Acetobacterium	0.09	(0.02, 0.36)	0.07	(0.02, 0.30)
Acidaminococcus	0.02	(0.00, 4.77)	0.00	(0.00, 9.34)
Akkermansia	0.06	(0.00, 31.54)	0.20	(0.00, 22.30)
Alkaliphilus	0.32	(0.04, 3.86)	0.43	(0.04, 6.37)
Anaerobranca	0.03	(0.00, 0.96)	0.06	(0.00, 2.17)
Anaerofilum	0.08	(0.00, 0.74)	0.12	(0.01, 1.35)
Anaerostipes	0.09	(0.00, 1.41)	0.08	(0.00, 2.65)
Bacteroides	16.13	(1.05, 57.72)	25.53	(4.52, 66.51)
Bifidobacterium	1.29	(0.08, 20.75)	1.04	(0.01, 13.84)
Bilophila	0.06	(0.00, 1.93)	0.12	(0.00, 1.06)
Blautia	6.76	(1.29, 18.49)	4.80	(0.00, 15.90)
Butyricimonas	0.04	(0.00, 0.59)	0.06	(0.00, 2.52)
Caldilinea	0.05	(0.00, 0.58)	0.08	(0.01, 0.650)
Caloramator	0.03	(0.01, 1.95)	0.07	(0.01, 14.09)
Candidatus Blochmannia	0.02	(0.01, 0.10)	0.03	(0.00, 2.87)
Catenibacterium	0.00	(0.00, 1.61)	0.00	(0.00, 4.67)
Clostridium	2.46	(0.74, 7.69)	2.17	(0.46, 10.65)
Collinsella	0.17	(0.00, 7.08)	0.08	(0.00, 2.14)
Coprococcus	0.30	(0.02, 4.44)	0.30	(0.04, 2.83)
Desulfonauticus	0.05	(0.00, 0.62)	0.07	(0.00, 1.27)
Desulfotomaculum	0.05	(0.00, 0.47)	0.07	(0.00, 1.50)
Desulfovibrio	0.08	(0.01, 9.54)	0.04	(0.00, 2.49)
Dialister	0.00	(0.00, 17.15)	0.01	(0.00, 15.22)
Dorea	0.27	(0.00, 5.27)	0.16	(0.00, 5.30)
Dysgonomonas	0.06	(0.00, 3.58)	0.04	(0.00, 12.40)
Enterobacter	0.07	(0.00, 2.67)	0.01	(0.00, 0.49)
Erysipelothrix	0.12	(0.02, 1.72)	0.16	(0.01, 7.34)
Escherichia	0.20	(0.00, 11.14)	0.04	(0.00, 1.80)

Table S5. Median and range of the relative abundance of each taxonomic level in OB and NW

	OB		NW	
**Taxonomic profile**	**Median**	**Range (min, max)**	**Median**	**Range (min, max)**
Eubacterium	0.02	(0.00, 2.12)	0.01	(0.00, 0.37)
Faecalibacterium	8.68	(0.42, 45.17)	6.10	(0.68, 37.40)
Flavobacterium	1.49	(0.02, 7.55)	3.42	(0.00, 21.82)
Gemella	0.08	(0.02, 0.48)	0.05	(0.00, 0.17)
Kitasatospora	0.08	(0.02, 1.22)	0.05	(0.01, 0.82)
Klebsiella	0.04	(0.00, 0.34)	0.00	(0.00, 10.64)
Lachnobacterium	0.01	(0.00, 2.97)	0.01	(0.00, 3.19)
Lachnospira	0.41	(0.04, 3.55)	0.47	(0.01, 4.68)
Lactobacillus	0.10	(0.01, 3.88)	0.08	(0.02, 1.02)
Luteibacter	0.00	(0.00, 0.04)	0.00	(0.00, 0.02)
Megamonas	0.00	(0.00, 13.29)	0.00	(0.00, 0.11)
Megasphaera	0.03	(0.00, 25.59)	0.01	(0.00, 0.24)
Mitsuokella	0.00	(0.00, 3.89)	0.00	(0.00, 0.20)
Natronincola	0.43	(0.11, 1.94)	0.28	(0.07, 1.35)
Negativicoccus	0.06	(0.00, 4.84)	0.01	(0.00, 0.27)
Oscillospira	1.58	(0.15, 18.28)	3.16	(0.00, 20.02)
Parabacteroides	0.88	(0.00, 6.29)	1.92	(0.08, 14.76)
Paraprevotella	0.06	(0.00, 14.47)	0.04	(0.00, 1.63)
Pedobacter	0.13	(0.00, 2.82)	0.44	(0.03, 3.89)
Peptoniphilus	0.07	(0.03, 0.75)	0.07	(0.00, 0.59)
Phascolarctobacterium	0.33	(0.03, 27.00)	0.97	(0.00, 7.47)
Prevotella	0.16	(0.01, 24.45)	0.04	(0.00, 39.80)
Pseudobutyrivibrio	0.31	(0.01, 5.04)	0.23	(0.00, 1.90)
Rikenella	0.02	(0.00, 4.27)	0.09	(0.00, 1.38)
Roseburia	1.75	(0.00, 52.25)	1.00	(0.00, 14.23)
Ruminococcus	3.50	(0.41, 13.09)	3.53	(0.00, 25.46)
Sedimentibacter	0.07	(0.01, 0.32)	0.07	(0.01, 0.94)
Serratia	0.09	(0.00, 5.70)	0.02	(0.00, 0.24)
Slackia	0.12	(0.03, 1.73)	0.06	(0.01, 1.18)
Sphingobacterium	0.09	(0.00, 0.54)	0.18	(0.00, 2.25)
Streptococcus	0.23	(0.05, 9.99)	0.10	(0.02, 1.45)
Sutterella	0.12	(0.01, 6.48)	0.39	(0.00, 2.61)
Thermicanus	0.03	(0.00, 3.45)	0.01	(0.00, 0.32)
Thiothrix	0.03	(0.00, 0.31)	0.06	(0.01, 0.78)
Veillonella	0.10	(0.01, 4.02)	0.03	(0.01, 1.79)
Acidaminococcus fermentans	0.00	(0.00, 2.95)	0.00	(0.00, 0.16)
Acidaminococcus intestini	0.01	(0.00, 1.90)	0.00	(0.00, 2.34)
Akkermansia muciniphila	0.06	(0.00, 31.54)	0.20	(0.00, 22.29)
Alkaliphilus crotonatoxidans	0.08	(0.00, 3.71)	0.22	(0.01, 6.31)
Alkaliphilus peptidifermentans	0.04	(0.00, 1.16)	0.02	(0.00, 4.40)
Anaerobranca zavarzinii	0.03	(0.00, 0.96)	0.06	(0.00, 2.17)
Anaerofilum pentosovorans	0.04	(0.00, 0.61)	0.06	(0.01, 1.23)
Bacteroides caccae	0.12	(0.00, 2.64)	0.43	(0.00, 4.51)
Bacteroides cellulosilyticus	0.03	(0.00, 0.79)	0.10	(0.00, 4.24)
Bacteroides coprocola	0.03	(0.00, 12.88)	0.01	(0.00, 11.67)
Bacteroides coprophilus	0.00	(0.00, 2.62)	0.00	(0.00, 3.51)
Bacteroides denticanum	0.10	(0.00, 3.91)	0.07	(0.01, 4.51)
Bacteroides dorei	0.31	(0.00, 18.13)	0.99	(0.00, 13.89)
Bacteroides eggerthii	0.00	(0.00, 13.40)	0.00	(0.00, 2.92)
Bacteroides fragilis	0.00	(0.00, 0.48)	0.00	(0.00, 4.28)
Bacteroides intestinalis	0.00	(0.00, 1.69)	0.00	(0.00, 3.61)
Bacteroides massiliensis	0.00	(0.00, 2.08)	0.00	(0.00, 1.57)
Bacteroides ovatus	0.13	(0.00, 7.41)	0.27	(0.00, 14.55)

Table S5. Median and range of the relative abundance of each taxonomic level in OB and NW

	OB		NW	
**Taxonomic profile**	**Median**	**Range (min, max)**	**Median**	**Range (min, max)**
Bacteroides paurosaccharolyticus	0.11	(0.00, 0.79)	0.13	(0.00, 0.70)
Bacteroides plebeius	0.00	(0.00, 8.26)	0.00	(0.00, 13.97)
Bacteroides rodentium	0.91	(0.043, 6.07)	2.29	(0.39, 13.66)
Bacteroides salanitronis	0.00	(0.00, 7.60)	0.00	(0.00, 1.48)
Bacteroides salyersiae	0.00	(0.00, 0.95)	0.00	(0.00, 9.30)
Bacteroides sartorii	0.16	(0.01, 4.03)	0.21	(0.02, 0.71)
Bacteroides stercorirosoris	0.27	(0.04, 2.02)	0.39	(0.11, 2.44)
Bacteroides stercoris	0.13	(0.00, 11.69)	0.08	(0.00, 20.16)
Bacteroides thetaiotaomicron	0.15	(0.00, 3.16)	0.30	(0.00, 6.49)
Bacteroides uniformis	0.80	(0.00, 6.21)	2.21	(0.31, 16.21)
Bacteroides vulgatus	0.68	(0.02, 20.48)	1.08	(0.04, 27.46)
Bacteroides xylanisolvens	0.64	(0.05, 6.14)	0.88	(0.14, 16.24)
Bifidobacterium adolescentis	0.05	(0.00, 7.55)	0.03	(0.00, 3.05)
Bifidobacterium catenulatum	0.01	(0.00, 7.30)	0.00	(0.00, 1.59)
Bifidobacterium choerinum	0.02	(0.00, 0.77)	0.02	(0.00, 0.85)
Bifidobacterium kashiwanohense	0.02	(0.00, 2.57)	0.00	(0.00, 0.250)
Bifidobacterium longum	0.12	(0.00, 7.76)	0.18	(0.00, 8.62)
Bifidobacterium scardovii	0.05	(0.00, 2.38)	0.06	(0.00, 1.64)
Bifidobacterium stercoris	0.20	(0.07, 5.58)	0.11	(0.00, 2.40)
Bilophila wadsworthia	0.05	(0.00, 1.92)	0.12	(0.00, 1.06)
Blautia coccoides	1.39	(0.25, 5.70)	1.01	(0.22, 5.07)
Blautia hansenii	0.29	(0.02, 1.36)	0.24	(0.02, 0.94)
Blautia obeum	0.01	(0.00, 0.51)	0.04	(0.00, 0.96)
Blautia wexlerae	0.32	(0.02, 5.90)	0.30	(0.04, 2.33)
Caldilinea tarbellica	0.05	(0.00, 0.58)	0.08	(0.01, 0.65)
Caloramator mitchellensis	0.02	(0.00, 1.93)	0.05	(0.00, 13.63)
Candidatus Blochmannia rufipes	0.00	(0.00, 0.00)	0.00	(0.00, 2.86)
Citrobacter freundii	0.00	(0.00, 4.98)	0.00	(0.00, 0.04)
Clostridium alkalicellulosi	0.31	(0.00, 2.55)	0.29	(0.03, 3.44)
Clostridium frigoris	0.07	(0.00, 2.23)	0.03	(0.00, 1.49)
Clostridium thermosuccinogenes	0.04	(0.00, 0.43)	0.05	(0.01, 0.48)
Collinsella aerofaciens	0.13	(0.00, 6.28)	0.05	(0.00, 1.82)
Coprococcus eutactus	0.00	(0.00, 2.46)	0.00	(0.00, 1.12)
Desulfonauticus autotrophicus	0.05	(0.00, 0.62)	0.07	(0.00, 1.27)
Desulfotomaculum indicum	0.05	(0.00, 0.47)	0.07	(0.00, 1.50)
Desulfovibrio fairfieldensis	0.01	(0.00, 8.00)	0.01	(0.00, 2.00)
Desulfovibrio piger	0.02	(0.00,1.04)	0.00	(0.00, 0.56)
Dialister invisus	0.00	(0.00, 17.14)	0.00	(0.00, 15.22)
Dorea formicigenerans	0.05	(0.00, 2.53)	0.04	(0.00, 0.70)
Dysgonomonas wimpennyi	0.06	(0.00, 3.58)	0.03	(0.00, 12.19)
Erysipelothrix inopinata	0.01	(0.00, 0.44)	0.04	(0.00, 3.35)
Erysipelothrix muris	0.08	(0.01, 1.72)	0.09	(0.01, 2.55)
Escherichia albertii	0.17	(0.00, 8.59)	0.03	(0.00, 1.78)
Eubacterium biforme	0.00	(0.00, 1.95)	0.00	(0.00, 0.36)
Faecalibacterium prausnitzii	1.41	(0.00, 6.71)	0.85	(0.00, 9.77)
Klebsiella variicola	0.01	(0.00, 0.04)	0.00	(0.00, 4.46)
Lachnospira pectinoschiza	0.26	(0.04, 2.25)	0.37	(0.01, 1.99)
Megamonas funiformis	0.00	(0.00, 4.86)	0.00	(0.00, 0.07)
Megasphaera hominis	0.02	(0.00, 8.38)	0.01	(0.00, 0.07)
Negativicoccus succinicivorans	0.06	(0.00, 4.84)	0.01	(0.00, 0.27)
Oscillospira eae	0.43	(0.03, 4.22)	0.74	(0.08, 7.12)
Oscillospira guilliermondii	0.05	(0.00, 0.44)	0.09	(0.03, 0.55)
Parabacteroides distasonis	0.24	(0.00, 3.39)	0.59	(0.00, 10.73)

	OB		NW	
**Taxonomic profile**	**Median**	**Range (min, max)**	**Median**	**Range (min, max)**
Parabacteroides goldsteinii	0.03	(0.00, 0.33)	0.05	(0.00, 3.81)
Parabacteroides johnsonii	0.04	(0.00, 4.23)	0.03	(0.00, 2.81)
Parabacteroides merdae	0.28	(0.00, 2.74)	0.49	(0.00, 4.70)
Paraprevotella clara	0.03	(0.00, 3.46)	0.03	(0.00, 1.42)
Pedobacter kwangyangensis	0.01	(0.00, 1.08)	0.03	(0.00, 2.14)
Phascolarctobacterium faecium	0.03	(0.01, 9.51)	0.01	(0.00, 3.98)
Phascolarctobacterium succinatutens	0.01	(0.01, 5.72)	0.12	(0.00, 2.75)
Prevotella copri	0.00	(0.00, 24.28)	0.00	(0.00, 26.17)
Prevotella shahii	0.01	(0.00, 1.86)	0.00	(0.00, 1.28)
Pseudobutyrivibrio xylanivorans	0.31	(0.01, 5.04)	0.23	(0.00, 1.90)
Rikenella microfusus	0.02	(0.00, 4.27)	0.09	(0.00, 1.38)
Roseburia faecis	0.55	(0.00, 6.19)	0.45	(0.00, 2.82)
Ruminococcus albus	0.00	(0.00, 1.61)	0.06	(0.00, 1.97)
Ruminococcus bromii	0.11	(0.00, 1.99)	0.30	(0.00, 6.69)
Ruminococcus callidus	0.02	(0.00, 0.97)	0.05	(0.00, 2.26)
Ruminococcus gnavus	0.32	(0.00, 9.82)	0.10	(0.01, 1.04)
Ruminococcus torques	0.03	(0.00, 2.23)	0.03	(0.00, 1.71)
Sphingobacterium bambusae	0.02	(0.00, 0.28)	0.02	(0.00, 2.22)
Sphingobacterium shayense	0.04	(0.00, 0.44)	0.09	(0.00, 1.23)
Streptococcus vestibularis	0.05	(0.00, 4.17)	0.02	(0.00, 0.61)
Sutterella wadsworthensis	0.01	(0.00, 5.90)	0.00	(0.00, 1.23)
Veillonella atypica	0.01	(0.00, 1.06)	0.01	(0.00, 0.57)

Medians were expressed as percentage (0-100), NW= normal-weight healthy controls, OB= obese and overweight patients.

Table S6. Firmicutes/Bacteroidetes ratio analysis between OB and NW

	Median (range)	*p*
		0.007
**OB**	1.77 (0.25, 21.90)	
**NW**	1.27 (0.17, 9.24)	

Differences in the Firmicutes/Bacteroidetes ratio between OB and NW, evaluated by Kruskal-Wallis test. NW= normal-weight healthy controls, OB= overweight and obese patients. *p*-value equal to or less than 0.05 was considered statistically significant.

Table S7. Firmicutes/Bacteroidetes ratio analysis in OB across different BMI categories

		Median (range)	*p*
			0.111
**NW**		1.12 (0.17, 9.24)	
**Overweight**		2.49 (1.01, 5.36)	
**OB**	**Class I**	1.70 (0.56, 18.96)	
	**Class II**	1.95 (0.25, 18.38)	
	**Class III**	2.55 (0.29, 21.90)	

Differences in the Firmicutes/Bacteroidetes ratio in OB across different BMI categories, evaluated by Kruskal-Wallis test. NW= normal-weight healthy controls, OB= overweight and obese patients. No statistically significant results were obtained in all the different categories. *p*-value equal to or less than 0.05 was considered statistically significant.

Table S8. Firmicutes/Bacteroidetes ratio analysis between NW, OWMS and OMS

	Median (range)	overall *p*	Bonferroni *p* (OMS *vs* NW)
		0.038	0.025
**NW**	1.12 (0.17, 9.24)		
**OWMS**	1.58 (0.56, 18.96)		
**OMS**	1.89 (0.25, 21.90)		

Differences in the Firmicutes/Bacteroidetes ratio, between NW, OMS and OWMS, evaluated by Kruskal-Wallis test followed by Bonferroni correction for multiple comparisons. NW= normal-weight healthy controls, OWMS= overweight and obese patients without metabolic syndrome, OMS= overweight and obese patients with metabolic syndrome. *p*-value equal to or less than 0.05 was considered statistically significant.

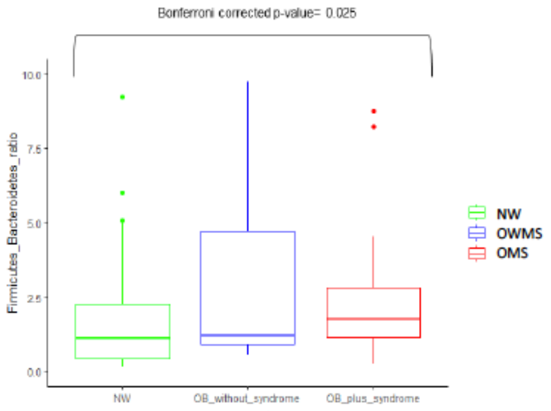

Fig. S2. Statistically significant differences in Firmicutes/Bacteroidetes ratio between NW, OWMS and OMS The statistical significance was observed only in the comparison between OMS and NW (*p*= 0.025) performed by Kruskal-Wallis test followed by Bonferroni correction for multiple comparisons in R software v.3.5.2. Plots were generated in R software v.3.5.2 (ggplot2 package). *p*-value equal to or less than 0.05 was considered statistically significant. NW= normal-weight healthy controls, OWMS= overweight and obese patients without metabolic syndrome, OMS= overweight and obese patients with metabolic syndrome.

Table S9. Median and range of the relative abundance of each statistically significant bacterial taxa in the comparison between OB and NW

Phylum	Family	Genus	Species	OB Median (range)	NW Median (range)
Bacteroidetes				29.69 (2.94, 74.72)	40.59 (7.18, 83.43)
Bacteroidetes	Bacteroidaceae	*Bacteroides*	*B. rodentium*	0.91 (0.04, 6.07)	2.29 (0.39, 13.66)
Bacteroidetes	Bacteroidaceae	*Bacteroides*	*B. uniformis*	0.80 (0.00, 3.16)	2.21 (0.31, 16.21)
Bacteroidetes	Flavobacteriaceae			1.61 (0.04, 7.65)	3.89 (0.18, 21.96)
Bacteroidetes	Flavobacteriaceae	*Flavobacterium*		1.49 (0.02, 7.55)	3.42 (0.00, 21.82)
Bacteroidetes	Porphyromonadaceae			1.16 (0.08, 6.86)	2.61 (0.09, 14.87)
Bacteroidetes	Rikenellaceae	*Rikenella*		0.02 (0.00, 4.27)	0.09 (0.00, 1.38)
Bacteroidetes	Rikenellaceae	*Rikenella*	*R. microfusus*	0.02 (0.00, 4.27)	0.09 (0.00, 1.38)
Bacteroidetes	Sphingobacteriaceae			0.38 (0.02, 3.45)	1.12 (0.15, 9.52)
Bacteroidetes	Sphingobacteriaceae	*Pedobacter*		0.13 (0.00, 2.82)	0.44 (0.03, 3.89)
Bacteroidetes	Sphingobacteriaceae	*Pedobacter*	*P. kwangyangensis*	0.01 (0.00, 1.08)	0.03 (0.00, 2.14)
Bacteroidetes	Sphingobacteriaceae	*Sphingobacterium*		0.08 (0.00, 0.54)	0.18 (0.00, 2.25)
Bacteroidetes	Tannerellaceae	*Parabacteroides*		0.88 (0.00, 6.29)	1.92 (0.08, 14.76)
Bacteroidetes	Tannerellaceae	*Parabacteroides*	*P. distasonis*	0.24 (0.00, 3.39)	0.59 (0.00, 10.73)
Firmicutes				54.00 (15.39, 87.86)	47.10 (13.85, 67.75)
Firmicutes	Acidaminococcaceae	*Acidaminococcus*	*A. intestini*	0.01 (0.00, 1.90)	0.00 (0.00, 2.34)
Firmicutes	Eubacteriaceae	*Eubacterium*	*E. biforme*	0.00 (0.00, 1.95)	0.00 (0.00, 0.36)
Firmicutes	Gemellaceae			0.08 (0.02, 0.48)	0.05 (0.01, 0.17)
Firmicutes	Gemellaceae	*Gemella*		0.08 (0.02, 0.48)	0.05 (0.00, 0.17)
Firmicutes	Lachnospiraceae			15.14 (3.35, 79.95)	10.39 (0.00, 26.07)
Firmicutes	Odoribacteraceae			0.12 (0.00, 0.68)	0.28 (0.00, 3.18)
Firmicutes	Paenibacillaceae			0.05 (0.01, 0.43)	0.03 (0.01, 0.36)

Table S9. Median and range of the relative abundance of each statistically significant bacterial taxa in the comparison between OB and NW

**Phylum**	**Family**	**Genus**	**Species**	**OB Median (range)**	**NW Median (range)**
Firmicutes	Ruminococcaceae	*Oscillospira*	*O. eae*	0.43 (0.03, 4.22)	0.74 (0.08, 7.12)
Firmicutes	Ruminococcaceae	*Ruminococcus*	*R. gnavus*	0.31 (0.02, 9.82)	0.10 (0.01, 1.04)
Firmicutes	Selenomonadaceae	*Megamonas*		0.00 (0.00, 13.29)	0.00 (0.00, 0.11)
Firmicutes	Selenomonadaceae	*Megamonas*	*M. funiformis*	0.00 (0.00, 4.86)	0.00 (0.00, 0.07)
Firmicutes	Selenomonadaceae	*Mitsuokella*		0.00 (0.00, 3.89)	0.00 (0.00, 0.20)
Firmicutes	Streptococcaceae			0.24 (0.06, 10.21)	0.10 (0.02, 1.48)
Firmicutes	Streptococcaceae	*Streptococcus*		0.23 (0.05, 9.99)	0.10 (0.02, 1.45)
Firmicutes	Thermicanaceae			0.03 (0.00, 3.45)	0.01 (0.00, 0.32)
Firmicutes	Thermicanaceae	*Thermicanus*		0.03 (0.00, 3.45)	0.01 (0.00, 0.32)
Firmicutes	Veillonellaceae	*Megasphaera*		0.03 (0.00, 25.59)	0.01 (0.00, 0.24)
Firmicutes	Veillonellaceae	*Megasphaera*	*M. hominis*	0.02 (0.00, 8.38)	0.01 (0.00, 0.07)
Firmicutes	Veillonellaceae	*Veillonella*		0.10 (0.01, 4.02)	0.03 (0.01, 1.79)
Firmicutes	Veillonellaceae	*Veillonella*	*V. atypica*	0.01 (0.00, 1.06)	0.01 (0.00, 0.57)
Proteobacteria	Alcaligenaceae			0.13 (0.01, 6.49)	0.39 (0.00, 2.62)
Proteobacteria	Desulfovibrionaceae	*Desulfovibrio*	*D. piger*	0.02 (0.00, 1.04)	0.00 (0.00, 0.56)
Proteobacteria	Enterobacteriaceae	*Candidatus Blochmannia*	*C.B. rufipes*	0.00 (0.00, 0.00)	0.00 (0.00, 2.86)
Proteobacteria	Enterobacteriaceae	*Enterobacter*		0.07 (0.00, 2.67)	0.01 (0.00, 0.49)
Proteobacteria	Enterobacteriaceae	*Escherichia*		0.20 (0.00, 11.14)	0.04 (0.00, 1.80)
Proteobacteria	Enterobacteriaceae	*Escherichia*	*E. albertii*	0.17 (0.00, 8.59)	0.03 (0.00, 1.78)
Proteobacteria	Enterobacteriaceae	*Klebsiella*		0.04 (0.00, 0.34)	0.00 (0.00, 10.64)
Proteobacteria	Sutterellaceae	*Sutterella*		0.12 (0.01, 6.48)	0.39 (0.00, 2.61)

Table S9. Median and range of the relative abundance of each statistically significant bacterial taxa in the comparison between OB and NW

Phylum	Family	Genus	Species	OB Median (range)	NW Median (range)
Proteobacteria	Yersiniaceae	*Serratia*		0.09 (0.00, 5.70)	0.02 (0.00, 0.24)

A multivariate association with linear models (MaAsLin) was used to perform a multivariate analysis on GM composition between OB and NW (adjusted for sex, age and smoking status) on R software v.3.5.2. Table shows only the statistically significant bacterial taxa in the comparison between OB and NW. NW= normal-weight healthy controls, OB= overweight and obese patients. Medians were expressed as percentage (0-100).

Table S10. Statistically significant differences in the relative abundance of bacterial taxa in OB across different BMI categories

Phylum	Family	Genus	Species	*q*	Coefficient	OB class III median (range)	NW median (range)
Bacteroidetes	Bacteroidaceae	*Bacteroides*	*B. uniformis*	0.18	0.11	0.31 (0.02, 1.08)	2.21 (0.31, 16.21)
Firmicutes	Erysipelotrichaceae	*Catenibacterium*		0.21	-0.01	0.06 (0.00, 0.63)	0.00 (0.00, 4.67)
Firmicutes	Selenomonadaceae	*Mitsuokella*		0.09	0.00	0.22 (0.00, 3.89)	0.00 (0.00, 0.20)
Proteobacteria	Enterobacteriaceae	*Candidatus Blochmannia*	*C. B. rufipes*	0.12	0.07	0.00 (0.00, 0.00)	0.00 (0.00, 2.86)

A multivariate association with linear models (MaAsLin) was used to perform a multivariate analysis on GM composition in OB across different BMI categories (adjusted for sex, age and smoking status) on R software (v.3.5.2). Statistically significant differences in the relative abundance of bacterial taxa were observed only in the comparison between OB class III group and NW. NW= normal-weight healthy controls, OB= overweight and obese patients, *q*-values: *p*-values adjusted for Benjamini and Hochberg False Discovery Rate (FDR) correction test for multiple comparisons (FDR<0.250), Coefficient= median difference between OB Class III and NW (median relative abundance in OB Class III minus median relative abundance in NW).

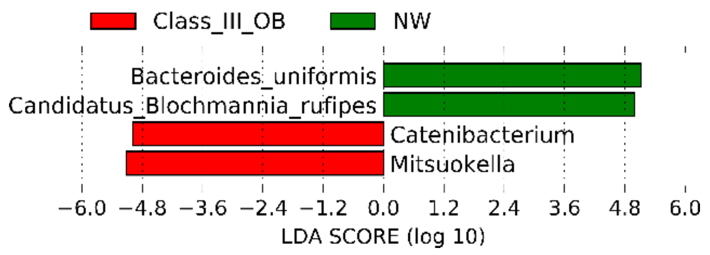

Fig. S3. Linear Discriminant Analysis Effect Size (LEfSe) of microbial taxa in OB across different BMI categories
LEfSe plot of taxonomic biomarkers was generated on Galaxy computational tool v.1.0. (https://huttenhower.sph.harvard.edu/galaxy/). Results were ranked by their Linear Discriminant Analysis (LDA) score. Red bacterial taxa were more abundant in OB Class III; green bacterial taxa were more abundant in NW. NW= normal-weight healthy controls, OB= overweight and obese patients

Table S11. Statistically significant differences in the relative abundance of bacterial taxa between OWMS and NW

Phylum	Family	Genus	Species	*q*	Coefficient	OWMS Median (range)	NW Median (range)
Firmicutes	Selenomonadaceae	*Megamonas*		0.001	-0.003	0.00 (0.01, 25.59)	0.01 (0.00, 0.24)
Firmicutes	Selenomonadaceae	*Megamonas*	*M. funiformis*	0.001	-0.003	0.00 (0.00, 0.01)	0.00 (0.00, 10.64)
Firmicutes	Veillonellaceae	*Megasphaera*		0.008	-0.005	0.03 (0.01, 25.59)	0.01 (0.00, 0.24)
Firmicutes	Veillonellaceae	*Megasphaera*	*M. hominis*	0.039	-0.005	0.03 (0.01, 8.38)	0.01 (0.00, 0.07)
Proteobacteria	Enterobacteriaceae	*Klebsiella*		-0.010	0.036	0.05 (0.00, 0.34)	0.00 (0.00, 10.64)

A multivariate association with linear models (MaAsLin) was used to perform a multivariate analysis on GM composition between OMWS and NW groups (adjusted for sex, age and smoking status) on R software (v.3.5.2). NW= normal-weight healthy controls, OWMS= overweight and obese patients without metabolic syndrome, *q*-values: *p*-values adjusted for Benjamini and Hochberg false discovery rate (FDR) correction test for multiple comparisons (FDR<0.05), Coefficient= median difference between OWMS and NW (median relative abundance in OWMS minus median relative abundance in NW).

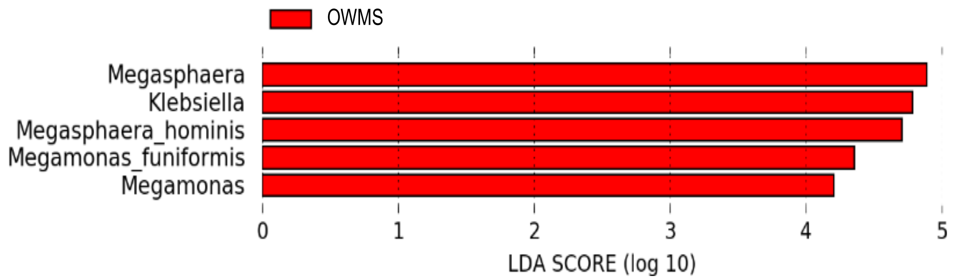

Fig. S4. Linear Discriminant Analysis Effect Size (LEfSe) of microbial taxa between OWMS and normal-weight controls
LEfSe plot of taxonomic biomarkers was generated on Galaxy computational tool v.1.0. (https://huttenhower.sph.harvard.edu/galaxy/). Results were ranked by their Linear Discriminant Analysis (LDA) score. Red bacterial taxa were more abundant in OWMS group compared to NW. NW= normal-weight healthy controls, OWMS= overweight and obese patients without metabolic syndrome.

Table S12. Statistically significant differences in the relative abundance of bacterial taxa between OMS and NW

**Phylum**	**Family**	**Genus**	**Species**	***q***	**Coefficient**	**OMS median (range)**	**NW Median (range)**
Firmicutes	Eubacteriaceae	*Eubacterium*	*E. biforme*	0.010	0.035	0.00 (0.00, 1.95)	0.00 (0.00, 0.36)
	Selenomonadaceae	*Megamonas*		0.004	0.003	0.00 (0.00, 13.29)	0.00 (0.00, 0.11)
			M. funiformis	0.004	0.003	0.00 (0.00, 4.86)	0.00 (0.00, 0.07)
	Veillonellaceae	*Megasphaera*		0.005	0.006	0.03 (0.00, 0.84)	0.01 (0.00, 0.24)
			M. hominis	0.010	0.005	0.02 (0.00, 0.33)	0.01 (0.00, 0.07)
Proteobacteria	Enterobacteriaceae	*Candidatus Blochmannia*	*C. B. rufipes*	0.004	-0.056	0.00 (0.00, 0.00)	0.00 (0.00, 2.86)
		Klebsiella		0.042	-0.014	0.03 (0.00, 0.25)	0.00 (0.00, 10.64)

A multivariate association with linear models (MaAsLin) was used to perform a multivariate analysis on GM composition between OMS and NW groups (adjusted for sex, age and smoking status) on R software (v.3.5.2). NW= normal-weight healthy controls, OMS= overweight and obese patients with metabolic syndrome, *q*-values: *p*-values adjusted for Benjamini and Hochberg false discovery rate (FDR) correction test for multiple comparisons (FDR<0.05), Coefficient= median difference between OMS and NW (median relative abundance in OMS minus median relative abundance in NW).

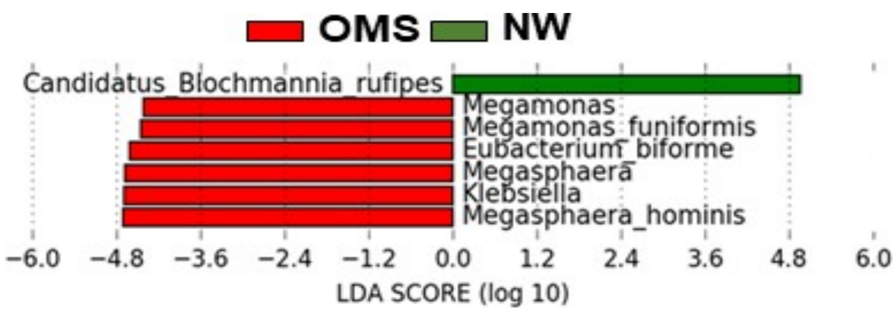

Fig. S5. Linear Discriminant Analysis Effect Size (LEfSe) of microbial taxa between OMS and normal-weight controls

LEfSe plot of taxonomic biomarkers was generated on Galaxy computational tool v.1.0. (https://huttenhower.sph.harvard.edu/galaxy/). Results were ranked by their Linear Discriminant Analysis (LDA) score. Red bacterial taxa were more abundant in OMS group; green bacterial taxa were more abundant in NW. NW= normal-weight healthy controls, OMS= overweight and obese patients with metabolic syndrome.

Table S13. Spearman's correlation between significant taxa and clinical parameters in OB

**Phylum**	**Family**	**Genus**	**species**	**Variable**	**R**	***p***
Bacteroidetes	Flavobacteriaceae			BMI	-0.399	0.006
				FM (%)	-0.365	0.013
				MedDietScore	0.307	0.038
				WC (cm)	-0.322	0.029
		Flavobacterium		FM (%)	-0.367	0.012
				MedDietScore	0.298	0.045
				WC (cm)	-0.325	0.028
	Odoribacteraceae			Age	0.345	0.019
	Odoribacteraceae			BMI	0.019	0.017
	Porphyromonadaceae			FM (%)	-0.292	0.049
	Rikenellaceae	*Rikenella*		Age	0.371	0.011
	Rikenellaceae	*Rikenella*	*R. microfusus*	Age	0.371	0.011
	Sphingobacteriaceae			MedDietScore	0.332	0.024
		Pedobacter		BMI	-0.297	0.045
				MedDietScore	0.308	0.037
		Pedobacter	*P. kwangyangensis*	BMI	-0.470	0.001
				FM (Kg)	-0.406	0.005
				MedDietScore	0.312	0.035
				WC (cm)	-0.414	0.004
	Tannerellaceae	*Parabacteroides*		Proteins (g)	0.311	0.045
Firmicutes	Selenomonadaceae	*Megamonas*		IPAQ (METs/weeks)	-0.342	0.020
		Megamonas		FM (%)	0.352	0.017
			M. funiformis	IPAQ (METs/weeks)	-0.304	0.045
	Thermicanaceae			MM (Kg)	-0.325	0.027
		Thermicanus		MM (Kg)	-0.325	0.027
	Veillonellaceae	*Megasphaera*		IPAQ (METs/weeks)	-0.342	0.020
			M. hominis	IPAQ (METs/weeks)	-0.365	0.013
Proteobacteria	Desulfovibrionaceae	*Desulfovibrio*	*D. piger*	MM (Kg)	-0.374	0.010

Spearman's correlations were calculated in R software (v.3.5.2). Only significant bacterial taxa identified in the multivariate analysis were correlated to clinical parameters. FM (%)= Fat Mass expressed in percentage, FM (kg)= kilograms of fat mass, IPAQ (METs/weeks) = number of METs for week, MM (kg)= Muscle Mass expressed in kg, WC= Waist circumference, BMI= Body Mass Index, MedDietScore= Mediterranean Diet Score, OB= obese and overweight patients. R= r coefficient. *p*-value equal to or less than 0.05 was considered statistically significant.

Table S14. Spearman's correlation between significant taxa and clinical parameters in NW

Phylum	Family	Genus	species	Variable	R	*p*
Bacteoidetes	Flavobacteriaceae	*Flavobacterium*		Proteins (g)	0.305	0.039
				IPAQ (METs/weeks)	0.304	0.040
		Sphingobacterium		Age	-0.362	0.013
Firmicutes	Eubacteriaceae	*Eubacterium*	*E. biforme*	Proteins (g)	0.299	0.043
				IPAQ (METs/weeks)	-0.397	0.006
	Veillonellaceae	*Megasphaera*		BMI	-0.364	0.013
	Veillonellaceae	*Megasphaera*	*M. hominis*	BMI	-0.319	0.031
	Enterobacteriaceae	*Candidatus Blochmannia*	*C. B. rufipes*	Age	-0.438	0.002

Spearman's correlations were calculated in R software (v.3.5.2). Only significant bacterial taxa identified in the multivariate analysis were correlated to clinical parameters, IPAQ (METs/weeks) = number of METs for week, BMI= Body Mass Index, NW= normal-weight healthy controls, R= r coefficient. *p*-value equal to or less than 0.05 was considered statistically significant.

References

1. Alberti KG, Zimmet P, Shaw J; IDF Epidemiology Task Force Consensus Group. The metabolic syndrome--a new worldwide definition. *Lancet*. Sep 24-30;**366**(9491):1059-62 (2005).
2. Panagiotakos, D. B., Pitsavos, C., Arvaniti, F., & Stefanadis, C. Adherence to the Mediterranean food pattern predicts the prevalence of hypertension, hypercholesterolemia, diabetes and obesity, among healthy adults; the accuracy of the MedDietScore. *Prev Med.* **44**(4), 335–340 (2007).
3. Lavelle, G. *et al*. Validity of the International Physical Activity Questionnaire Short Form (IPAQ-SF) as a measure of physical activity (PA) in young people with cerebral palsy: A cross-sectional study. *Physiotherapy*. **107**, 209–215 (2020).
